# Gadolinium‐containing carbon nanomaterials for magnetic resonance imaging: Trends and challenges

**DOI:** 10.1111/jcmm.15065

**Published:** 2020-03-10

**Authors:** Andrés Rodríguez‐Galván, Margarita Rivera, Patricia García‐López, Luis A. Medina, Vladimir A. Basiuk

**Affiliations:** ^1^ Unidad de Investigación Biomédica en Cáncer INCan‐UNAM Instituto Nacional de Cancerología Ciudad de Méxi Mexico; ^2^ Carrera de Biología Unidad de Biomedicina Facultad de Estudios Superiores Iztacala Universidad Nacional Autónoma de México Tlalnepantla Mexico; ^3^ Instituto de Física Universidad Nacional Autónoma de México Coyoacán Ciudad de México Mexico; ^4^ Laboratorio de Farmacología Subdirección de Investigación Básica Instituto Nacional de Cancerología Ciudad de México Mexico; ^5^ Instituto de Ciencias Nucleares Universidad Nacional Autónoma de México Ciudad de México Mexico

**Keywords:** biodistribution, carbon nanomaterials, cell labelling, gadolinium contrast agent, magnetic resonance imaging, multifunctional contrast agent, toxicity

## Abstract

Gadolinium‐containing carbon nanomaterials are a new class of contrast agent for magnetic resonance imaging. They are characterized by a superior proton relaxivity to any current commercial gadolinium contrast agent and offer the possibility to design multifunctional contrasts. Intense efforts have been made to develop these nanomaterials because of their potential for better results than the available gadolinium contrast agents. The aim of the present work is to provide a review of the advances in research on gadolinium‐containing carbon nanomaterials and their advantages over conventional gadolinium contrast agents. Due to their enhanced proton relaxivity, they can provide a reliable imaging contrast for cells, tissues or organs with much smaller doses than currently used in clinical practice, thus leading to reduced toxicity (as shown by cytotoxicity and biodistribution studies). Their active targeting capability allows for improved MRI of molecular or cellular targets, overcoming the limited labelling capability of available contrast agents (restricted to physiological irregularities during pathological conditions). Their potential of multifunctionality encompasses multimodal imaging and the combination of imaging and therapy.

## 1 INTRODUCTION

Magnetic resonance imaging (MRI) is one of the most important tools for clinical imaging. Apart from its unlimited tissue penetration, MRI offers the advantages of being non‐invasive and producing zero ionizing radiation. Because of being able to distinguish tumours from healthy tissue, it is considered essential for the diagnosis and prognosis of cancer as well as for surveying the efficacy of the pharmacotherapy.^1^ Additionally, it is used for the preparation and guidance of surgical procedures (eg brain tumour resections)^2^ and for basic biomedical research.

An MRI of the human body is based on signals produced by water protons under a strong magnetic field.^3^ Since most of the human body is made up of water molecules, MRI can identify a given organ or tissue structure by detecting water proton density, proton velocity, and the longitudinal (T1) and transverse (T2) relaxation times.^4^ When the intrinsic contrast between diseased and healthy tissues is too low for an accurate diagnosis,^5^ contrast agents are intravenously administered. These chemical entities improve the visualization of tissues or organs by increasing the relative difference between the signal intensity.^6^ They improve MRI contrast by shortening the longitudinal (T1) and/or transverse (T2) relaxation times of water protons.

MRI contrast agents are classified as T1 or T2 according to the type of relaxation time that is improved. T1 predominantly reduces the longitudinal relaxation time of water protons, thus enhancing a positive (bright) contrast in imaging. Contrarily, T2 primarily diminishes the transverse relaxation time, improving the negative (dark) contrast.^7^, ^8^ Regarding the T1‐type, the main medical imaging agents for the visualization of tissues and organs contain gadolinium(III) [Gd(III)]. This metal ion couples a large magnetic moment with a long electron spin relaxation time.^9^ Although Gd(III) complexes have been administered to more than 100 million patients and demonstrated an extraordinarily positive safety record,^10^ it is necessary to develop new contrast agents that improve diagnosis accuracy.^11^

**Figure 1:**
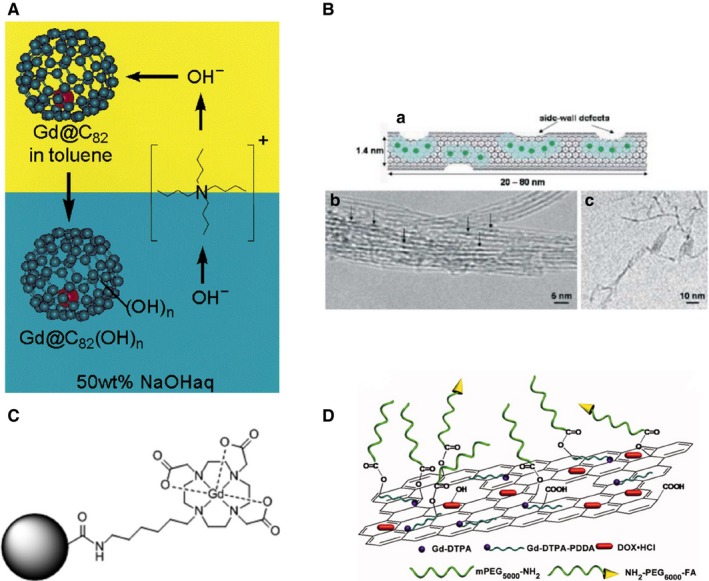
Examples of gadolinium‐containing carbon nanomaterials. (A) Scheme of the synthesis of water‐soluble polyhydroxylated gadofullerenes (Gd@C_82_(OH)_n_). Reprinted with permission from,^12^ copyright 2001 American Chemical Society. (B) Images of gadonanotubes. (a) Depiction of a single US‐tube loaded with hydrated Gd^3+^ ions and its (b) HRTEM and (c) Cryo‐TEM images. Reprinted with permission from,^13^ copyright 2005 Royal Society of Chemistry. (C) Structural drawing of a gadonanodiamond. Gd(III) complexes are employed for nanodiamond functionalization. Reprinted with permission from,^37^ copyright 2010 American Chemical Society. (D) Scheme of the structure of a gadographene: GO‐PEG‐FA/Gd/DOX. DOX, doxorubicin; DTPA, diethylenetriamine‐pentaacetic acid; FA, folic acid; GO, graphene oxide; PEG, polyethylene glicol. Reprinted with permission from,^38^ copyright 2012 Wiley & Sons

Gadolinium‐containing carbon nanomaterials, a new class of T1 contrast agents, rank among the most potent in terms of proton relaxivity (*r*_1_). They induce 10‐ to 90‐fold greater relaxivity^12^, ^13^ than the available gadolinium‐based agents. For example, the relaxivity induced by Gd(DTPA) or Gd(DOTA) is at about 4 mM^−1^ s^−1^ while for gadofullerenes is around *r*_1_ = 47.0 ± 1.0 mM^−1^ s^−1^.^11^ The new gadolinium contrast consists of carbon nanomaterials with gadolinium ions confined in their inner cavities or attached by ligands to functional groups on their external surface, tips or edges. The Figure 1 shows the principal four types of contrast agents: gadofullerenes, gadonanotubes, gadonanodiamonds and gadographene.

Compared to commercial products, the new contrast agents show several advantages. Recent reviews have discussed the advances of gadolinium‐containing carbon nanomaterials in relation to the methods of synthesis as well as their chemical and physical properties. Each review has focused on a certain type of nanomaterial, such as gadonanotubes^14^, ^15^, ^16^ or gadofullerenes.^17^, ^18^, ^19^, ^20^, ^21^, ^22^ The present review consists of an overview of gadolinium‐containing carbon nanomaterials, comparing them with conventional gadolinium contrast agents and considering their advantages, disadvantages, cytotoxicity and biodistribution.

## 2 POTENTIAL ADVANTAGES

**Figure 2:**
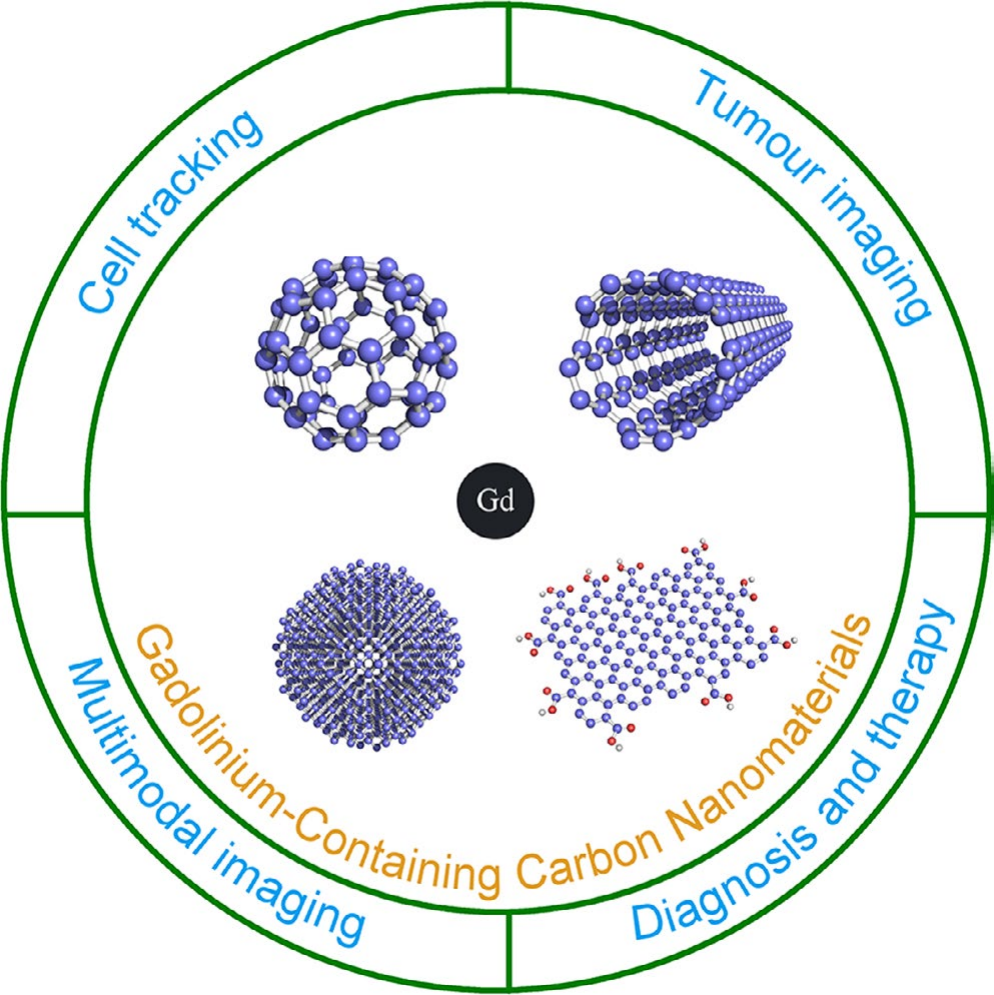
Schematic illustration of the major biological applications of gadolinium‐containing carbon nanomaterials. The chemical and physical properties of carbon nanomaterials let the design of new MRI contrast agents for multimodal imaging, cell tracking, tumour imaging and the combination of diagnosis and therapy

There are several important advantages afforded by gadolinium‐containing carbon nanomaterials compared to commercial contrast agents (Figure 2). Firstly, their greater proton relaxivity allows for the attainment of reliable images with a lower dose. Hence, less Gd(III) ions are needed (<10‐fold less than the current clinical dose) to produce the same brightness as available contrast agents.^23^ Secondly, specific cells, tissues and molecules can be targeted. Contrast selectivity is achieved by the modification of carbon nanomaterials with biopolymers such as DNA, RNA, peptides and proteins.^24^, ^25^, ^26^, ^27^, ^28^ Thirdly, carbon nanomaterials are able to translocate across membranes and label cells. Their excellent cell penetration likely results from their size, aspect, ratio and amphiphilic surface character.^29^, ^30^, ^31^ Fourthly, these carbon nanomaterials have multifunctional potential. Their optical properties permit the design of contrast agents capable of multimodal imaging.^32^ Their large surface area and chemically tailorable surface enable molecules to be efficiently loaded by covalent and non‐covalent functionalization for a combination of imaging and therapy.^32^, ^33^, ^34^

### 2.1 Reduced amount of the contrast agent required to obtain reliable images

**Figure 3:**
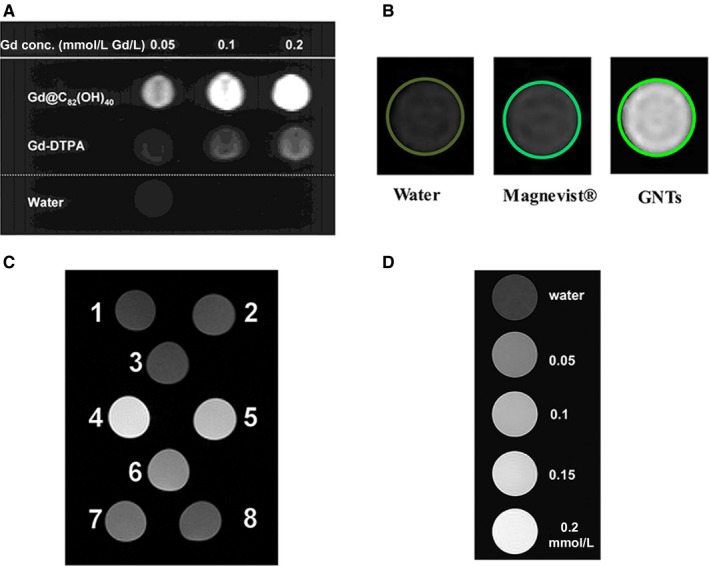
Improved relaxivity of gadolinium‐loaded carbon nanomaterials. (A) T1‐weighted MRI of Gd@C_82_ (OH)_40_ and the Gd‐DTPA phantom illustrate that with an equivalent concentration of gadolinium and the gadofullerenes, the latter had the strongest signal. Reprinted with permission from,^12^ copyright 2001 American Chemical Society. (B) T1‐weighted magnetic resonance imaging (MRI) phantoms of Gd^3+^n@US‐tubes and Magnevist™ at the same concentration; pure water is also shown. Reprinted with permission from,^48^ copyright 2017, the Electrochemical Society. (C) MR images of nanodiamond samples. 1, water; 2, nanodiamonds; 3, nanodiamonds + coupling reagents. Gadonanodiamonds at different concentrations of gadolinium: 4, 48 μmol/L Gd(III); 5, 38 μmol/L Gd(III); 6, 22 μmol/L Gd(III); 7, 10 μmol/L Gd(III); 8, 5 μmol/L Gd(III). Reprinted with permission from,^37^ copyright 2010 American Chemical Society. (D) T1‐weighted MR images of pure water and gadographene solutions at various concentrations. Reprinted with permission from,^31^ copyright 2018 Elsevier Ltd

Gadolinium‐containing carbon nanomaterials have been consistently found to induce high proton relaxivity. In 1997, Zhang et al^35^ reported that gadofullerenes produce a nearly 10‐fold greater proton relaxivity than current contrast agents (*r*_1_ = 47.0 ± 1.0 mM^−1^ s^−1^), including Gd(DTPA) (*r*_1_ = 4.30 mM^−1^ s^−1^), Gd(DOTA) (*r*_1_ = 4.20 mM^−1^ s^−1^), Gd(DTPA‐BMA) (*r*_1_ = 4.39 mM^−1^ s^−1^) and Gd(HP‐D03A) *r*_1_ = 3.70 mM^−1^ s^−1^). Therefore, they proposed the use of gadofullerenes as a contrast agent for MRI.^36^ A few years later, they documented the first T1‐weighted MRI phantoms obtained with gadofullerenes and carried out the first in vivo studies on the viability of gadofullerenes as contrast agents^12^ (Figure 3A). In 2005, Sitharaman et al^13^ demonstrated a threefold greater relaxivity of gadonanotubes (*r*_1_ = 170 mM^−1^ s^−1^) compared to gadofullerenes. Soon after, the same group published the first T1‐weighted MRI phantoms of gadonanotubes^14^ (Figure 3B). In 2010, Manus et al^37^ found a nearly 10‐fold greater relaxivity of gadonanodiamonds (*r*_1_ = 58.82 ± 1.18 mM^−1^ s^−1^) compared to the current contrast agents. They reported the first T1‐weighted MRI phantoms with gadonanodiamonds (Figure 3C). In 2012, Shen et al^38^ showed the high relaxivity produced by gadographene, demonstrated by the first T1‐weighted MRI phantoms with this contrast agent (Figure 3D).

Although the working mechanism for the superior relaxivity of gadolinium‐containing carbon nanomaterials is still not well understood, it is often described by the Solomon, Bloembergen and Morgan (SBM) theory. This theory, initially developed to explain the proton relaxivity of gadolinium chelated contrasts, provides insights into gadolinium‐containing carbon nanomaterials. Several parameters of the SBM theory can help to account for the observed high relaxivity of the new carbon contrast. These include the number of water molecules co‐ordinated to the paramagnetic centre (*q*), the rotational correlation time (TR) and the mean residence life‐time of the co‐ordinate waters (TM).

**Figure 4:**
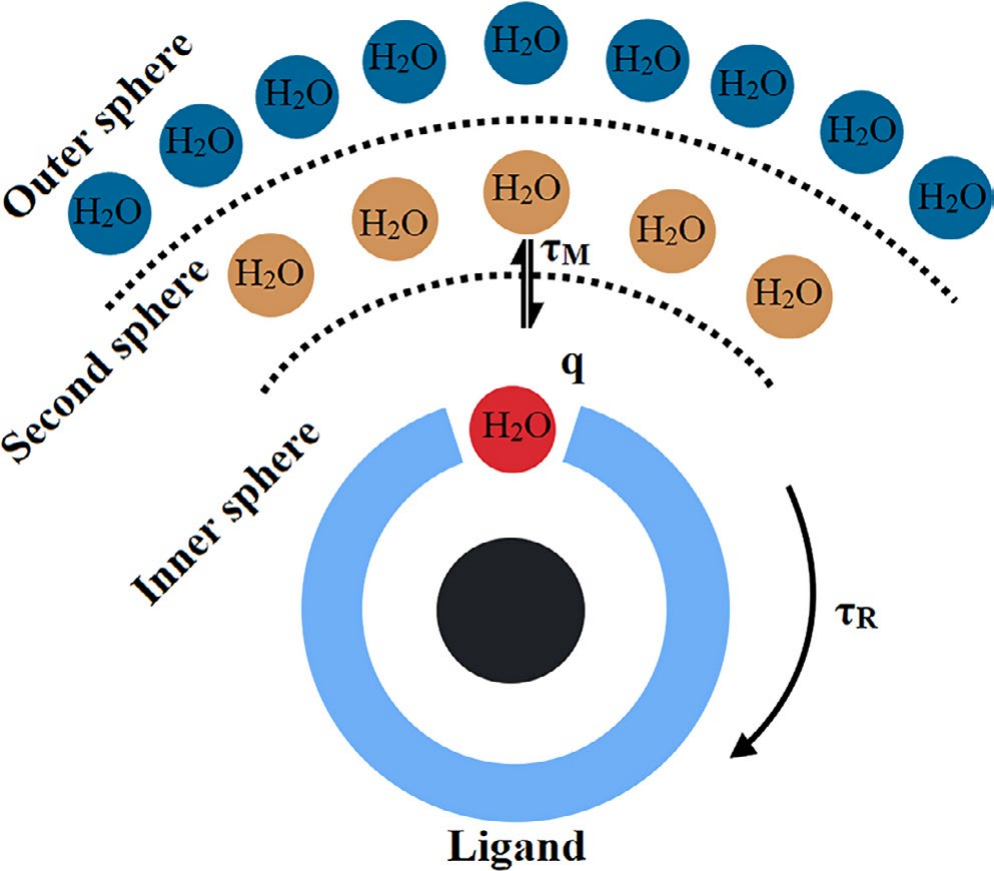
Three different types of water molecules hydrating a Gd(III) complex. The inner sphere directly co‐ordinated to the Gd ion is represented by the red circle; the second‐sphere forming hydrogen bonds with the complex is portrayed by the brown circles; the outer sphere is depicted by the blue circles

The number of water molecules co‐ordinated is considered one of the most important. Gd(III) is a paramagnetic element with a fluctuating magnetic field that interacts with nearby water molecules and thus shortens their T1 and T2 relaxation times and improves the bright contrast in MR imaging.^39^ The Gd(III) complex is hydrated by three different types of water molecules that may be affected by the magnetic field (Figure 4). According to the SBM theory, proton relaxivity enhancement is more pronounced for water molecules directly co‐ordinated to the paramagnetic centre (in the inner sphere) than those at a greater distance (in the second and outer sphere). These water molecules play a key role in transmitting the paramagnetic effect towards the bulk solvent and therefore in improving the contrast. Commercial Gd(III) contrast agents usually have one co‐ordinate water molecule (*q* = 1) and a relaxivity of approximately of four. For example, Gd(DTPA) displays an *r*_1 _value of 4.30 mM^−1^ s^−1^. Gadonanotubes, on the other hand, reportedly contain a large number of co‐ordinated water molecules per Gd(III) (*q* = 9), as suggested by X‐ray absorption spectroscopic analysis,^40^ which may explain their elevated relaxivity compared to commercial gadolinium chelated contrast agents. The *q* value has also been employed to account for the superior performance of gadofullerenes vs commercial agents.^12^ The paramagnetic properties of Gd(III) are considered to be conserved and transferred to the fullerene cage in gadofullerenes, where the Gd(III) ion is encapsulated.^41^, ^42^ Consequently, there is an enhanced surface area accessible to water molecules, allowing a larger number of them to have direct contact with the paramagnetic centre and undergo the transmission of relaxivity. For gadofullerenes and gadonanotubes, it was reported a second sphere‐like mechanism also plays a key role.^43^ This second‐sphere like is originated by a large number of water molecules bounded to the functional groups present in the carbon nanostructure surface, which increases the probability of waters protons to be exchanged.^44^, ^45^ The SBM theory also consider the rotational correlation time (TR), which refers to the tumbling rate of the contrast agents.^39^ The optimal TR value for a boosted relaxivity is in the range of nanoseconds.^37^ Small gadolinium contrasts agents tumble too fast, near the tenths of picoseconds, and in order to decrease their tumbling rate they are usually coupled to polymers of high molecular weight.^35^ Magnetic relaxation dispersion profiles (NMRD) and dynamic light scattering (DLS) analysis have shown the size of the gadofullerenes and their aggregation state are linked with their rotational correlation time. Laus et al^46^ reported the disaggregation of Gd@C_60_(OH)_x_ and Gd@C_60_[C(COOH_2_)]_10_ by salt addition leads to more rapidly tumbling rates and lower relativities. Tóth et al^47^ found the TR of Gd@C_60_[C(COOH)_2_]_10_ decrease when it is disaggregated, from TR = 2.6 ns to TR = 1.2 ns. In the case of gadonanotubes, NMRD studies have shown, gadonanotubes have relaxation profiles characteristic of gadolinium ions co‐ordinated to a slowly tumbling environment.^48^

On the other hand, it was found the carbon structure also plays a key role for water proton relaxation. Moghaddam et al,^48^ found the carbon‐based radical centres that are supported by the polynuclear aromatic structure contribute at low magnetic fields (<1 MHz).

Until now, several parameters have described the relaxivity of gadolinium‐containing carbon materials, however, there is a necessity to develop new theoretical approaches for a most robust description [for a more comprehensive review, see^15^, ^40^, ^49^, ^50^].

One of the most important implications of the heightened relaxivity of gadolinium‐containing carbon nanomaterials compared to conventional contrast agents is the reduction of the dose of Gd(III) required to evaluate areas of clinical interest (Figure 3). For example, Mikawa et al^12^ found the MRI signal to be stronger for polyhydroxylated gadofullerenes than Gd‐DTPA when both had an equivalent concentration of Gd(III). Li et al^23^ pointed out that the concentration of Gd‐DTPA should be 50‐fold higher than amino functionalized gadofullerenes in order to achieve the same contrast. Fatouros et al^51^ obtained equivalent contrast intensity with pegylated‐hydroxylated gadofullerenes and gadodiamide (Omniscan), even though the latter had to be administered at a 30‐fold higher concentration. Sitharaman et al^14^ applied gadonanotubes and Magnevist at the same concentration and observed an ~100‐fold higher signal intensity for the former.

### 2.2 Easy targeting of the contrast agent towards specific cells, tissues or molecules

Nowadays, research on non‐invasive imaging focuses on the development of tools for detecting biomarkers, which are unique biochemical signatures that differentiate and characterize tissues in a diseased state at the cellular or molecular level. MRI provides high‐resolution images to evaluate anatomical structures but has limited sensitivity to visualize biomarkers. MRI images of diseased tissues are generally formed by the accumulation of the contrast agent at sites with histological irregularities. For example, due to the abundant vascularization existing in brain tumours and the consequent presence of pores in the blood‐brain barrier, contrast agents can diffuse freely and accumulate in the tumour, thereby facilitating MR imaging.^52^ Contrarily, contrast agents are not able to cross the blood‐brain barrier in healthy tissue.

Another disadvantage of MRI is the inability of most contrast agents to cross intact membranes, restricting them to the extracellular space and thus making it impossible for them to target intracellular biomarkers.^53^ To overcome these disadvantages, scientist has focus on the development of new contrast agents to improve the MRI potential for the detection of biomarkers at the cellular or molecular level. In particular, the efforts have been focused on ‘active targeting’ concept, which involve the binding of certain ligands to contrast agents for specific homing^54^ The ligands are selected based on their ability to bind to biomarkers in the target cells (eg membrane receptors overexpressed on the surface of cancer cells), allowing for the uptake and retention of the contrast agent and as a consequence the imaging of the molecular targets.

The active targeting concept inspired the development of gadolinium‐based carbon nanomaterials, in which single amino acids, small peptides, cyclic peptides, proteins and organic molecules are integrated as ligands to target specific cells. For instance, Shu et al^26^ worked with antibodies able to recognize the green fluorescent protein. These antibodies were bound to carboxylic functional groups of gadofullerenes and the authors suggested that this strategy can be extended to other biomolecules or endohedral fullerenes.

**Figure 5:**
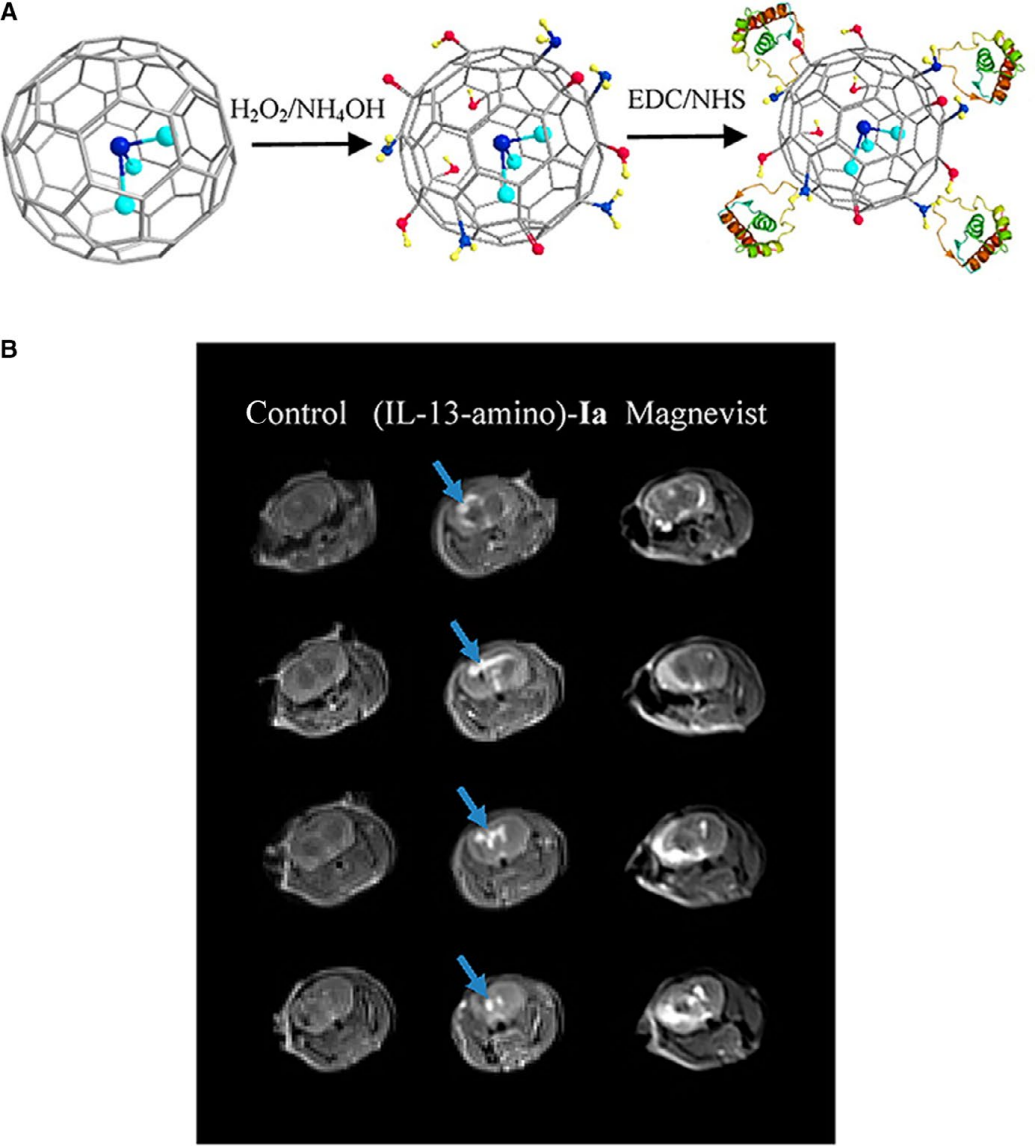
(A) Scheme of functionalization and conjugation process of Gd_3_N@C_80_ with the interleukin‐13 (IL‐13‐amino)‐Ia. (B) In vivo imaging following intravenous delivery of (IL‐13‐amino)‐Ia. Left: MRI of a mouse brain without tumour (control). Middle: MRI of a mouse brain 15 min after intravenous (iv) injection of 300 μL (~0.9 nmol) of (IL‐13‐amino)‐Ia. The bright contrast is due to the presence of (IL‐13‐amino)‐Ia. Right: MRI of a mouse brain 15 min post iv injection of 100 μL (50 nmol) of Magnevist commercial contrast agent. Arrows indicate the location of the tumours. Reprinted with permission from,^23^ copyright 2015 American Chemical Society

Fillmore et al^27^ reported the covalent binding of the cytokine interleukin‐13 (IL‐13) peptide to the carboxylic groups on gadofullerenes. They selected IL‐13 because of its high affinity for the IL‐13Rα2 receptor, which is overexpressed on human glioma cells. When comparing gadofullerenes with and without the IL‐13 functionalization, the former displayed a 64‐fold greater in vitro uptake. The authors also actively targeted a tumour in mouse brain (implanted by the inoculation of U87 cells) with functionalized gadofullerenes and could achieve MR imaging. Years later, they actively targeted a brain tumour with gadofullerenes bearing amino groups (rather than carboxylic groups) for the covalent binding of IL‐13.^23^ This conjugate system produced images with a sharp definition of the tumour, unlike the low‐quality tumour outline obtained with Magnevist (the control) (Figure 5). Since active targeting improved the capacity of MRI to detect tumours, the concentration employed was up to 50% lower than that of traditional control contrast agents.^23^

**Figure 6:**
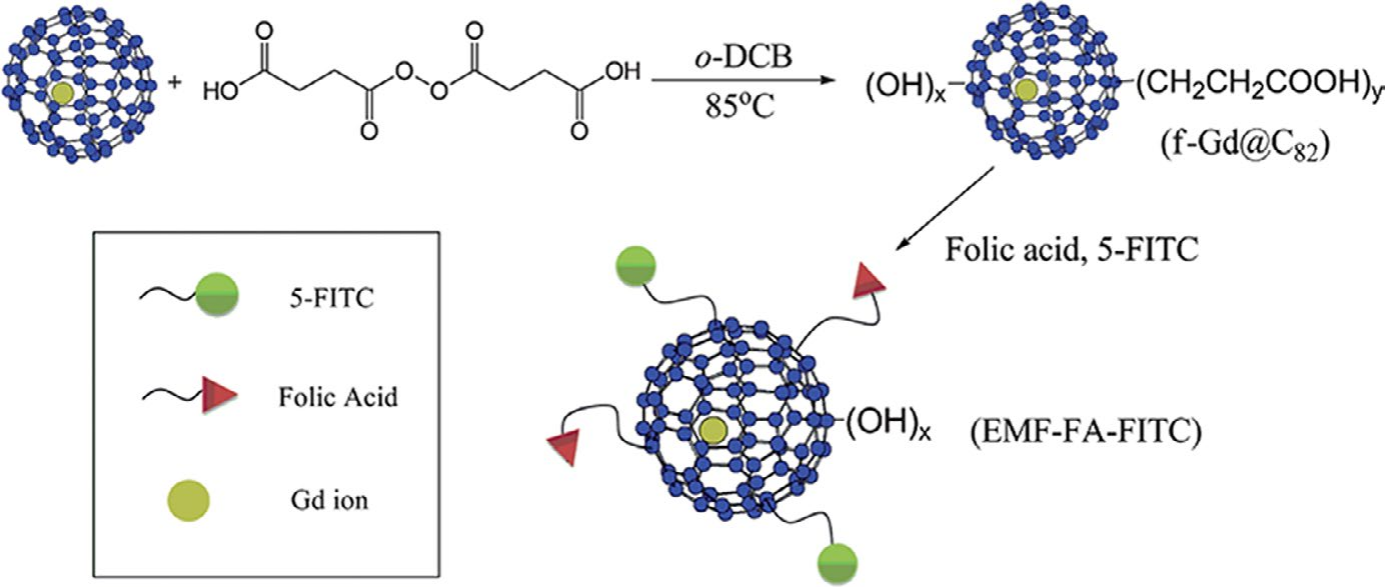
Scheme of the synthesis of gadofullerenes functionalized with folic acid. Reprinted with permission from,^55^ copyright 2012 Royal Society of Chemistry

Gadofullerenes have been modified with folic acid as well, a ligand with high affinity for its cell membrane receptor, which is overexpressed on the surface of many types of cancer cells (lung, ovary, breast, brain, kidney, colon and endometrium). For instance, Zheng et al^55^ compared unmodified gadofullerenes to those functionalized with folic acid (Figure 6), finding a preferential uptake of the latter by cancer cells under in vitro conditions.

**Figure 7:**
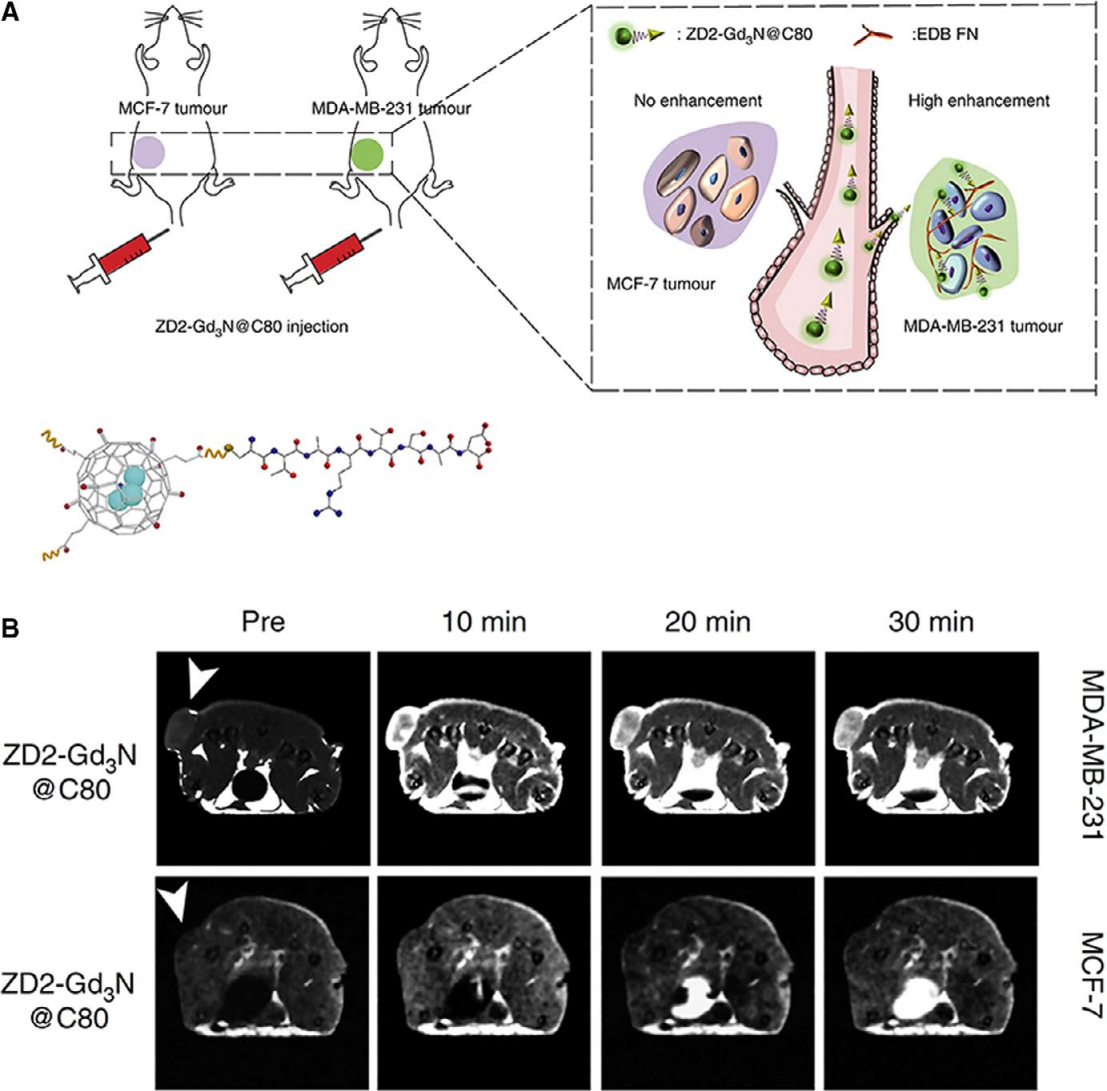
Functionalized gadofullerenes developed for the detection of breast cancer. (A) Scheme of the functionalized gadofullerenes ZD2‐Gd_3_N@C_80_ and their tumour targeting capability. The MCF‐7 and MDA‐MB‐231 cells lines were used to obtain low‐risk and high‐risk breast cancer xenografts, respectively. (B) The functionalized gadofullerenes afforded a specific MRI detection of high‐risk breast cancer xenografts. Tumour locations are indicated with white arrow heads. Reprinted with permission from reference,^25^ copyright 2017 Nature Publishing Group (http://creativecommons.org/licenses/by/4.0/)

More recently, Han et al^25^ explored the active targeting of extracellular matrix proteins. This strategy emerged as alternative due to the difficulties to target cancer cells and is based in findings that have shown certain proteins are abundantly expressed in regions surrounding tumours and at basal levels in healthy tissues.^56^, ^57^ Han et al functionalized gadofullerenes with the small peptide ZD2 (Cys‐Thr‐Val‐Arg‐Thr‐Ser‐Ala‐Asp), which has high affinity towards the extra domain‐B fibronectin (EDB‐FN), a protein abundantly expressed in the extracellular matrix of many types of aggressive human cancers (Figure 7). Hence, these tumours can be targeted in vitro and in vivo, facilitating their detection with MRI.

In summary, specific functionalization of gadofullerenes with high‐affinity ligands by biomarkers has enabled the active targeting of tumours at a cellular and molecular level, leading to increased MRI capability.

### 2.3 Potential for the design of multifunctional nanoprobes

**Figure 8:**
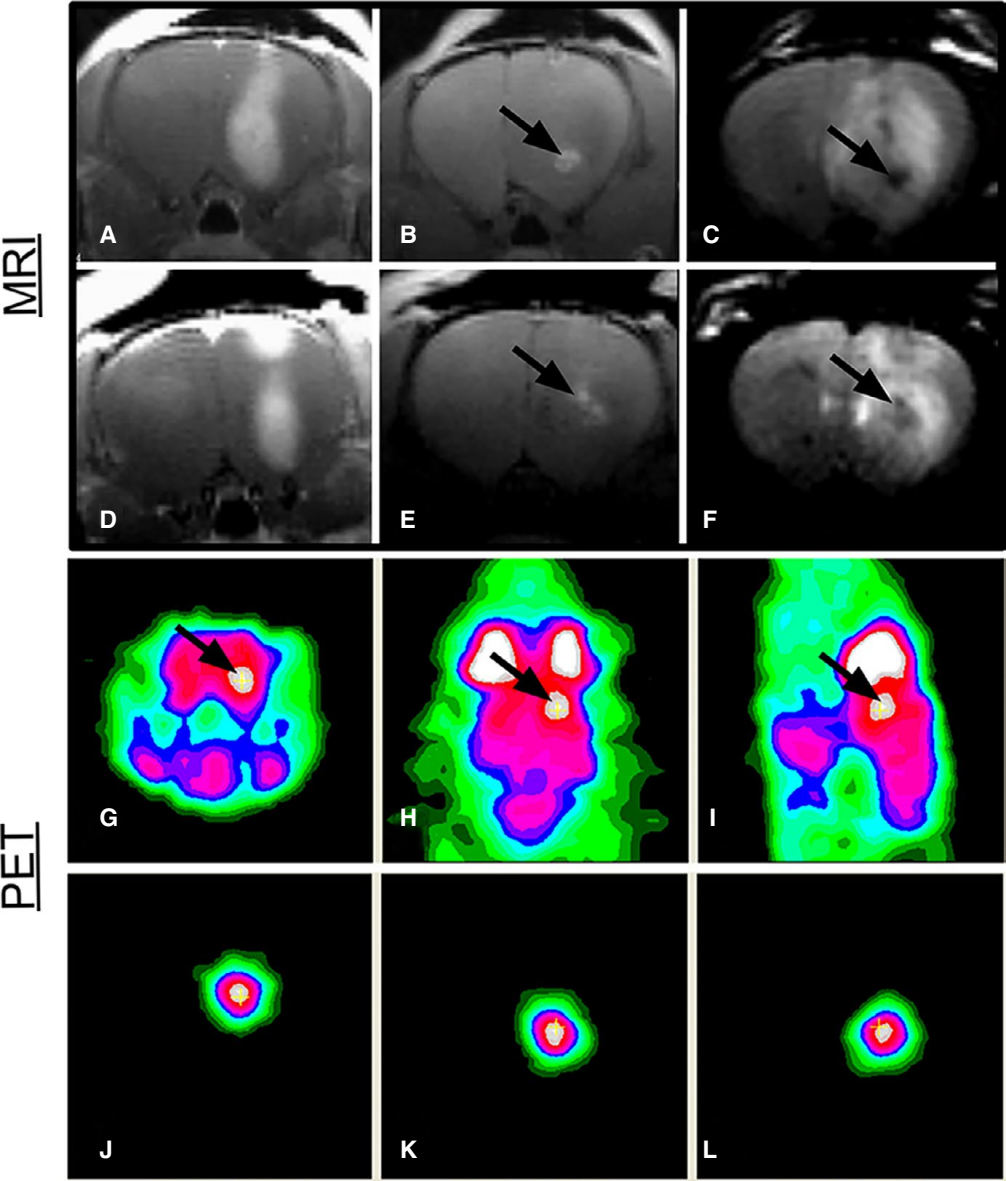
Gadofullerenes developed for multimodal imaging detection of brain tumours. (A, D) T1‐weighted images with gadodiamide contrast. (B, E) T1‐weighted images in which the bright contrast at the infusion site results from ^124^I‐f‐Gd_3_N@C_80_. (C, F) T2‐weighted images with dark contrast due to ^124^I‐f‐Gd_3_N@C_80_. (G) Coronal, (H) axial and (I) sagittal microPET images following ^18^F‐FDG injection and the additive image signal that allows for localization of ^124^I‐f‐Gd_3_N@C_80_ within the right hemisphere of the rat brain (arrows indicate infusion sites). (J‐L) MicroPET images display the signal from ^124^I‐f‐Gd_3_N@C_80_. Reprinted with permission from,^62^ copyright 2012 MDPI (http://creativecommons.org/licenses/by/4.0/)

A multifunctional probe is a contrast agent that serves multiple imaging modes or is able to facilitate imaging and therapy at the same time. Although current small gadolinium‐based contrast agents have the possibility of multifunctionality, their chemical function is complex and multiple steps are required for their synthesis. In contrast, carbon nanomaterials require minimal steps to increase their multifunctionality. For instance, it is possible, in the absence of a chemical reaction, to non‐covalently functionalize carbon nanomaterials with small organic molecules, organic polymers or biomolecules.^33^, ^34^, ^58^, ^59^ Taking advantage of this several multifunctional contrast agents had developed. For instance, Cisneros et al^60^ developed gadonanotubes in the absence of a chemical reaction, to be used for imaging with MRI and positron emission tomography (PET). They loaded ultra‐short single‐walled carbon nanotubes with both Gd(III) and ^64^Cu(II) ions to form the conjugate ^64^Cu(II)@gadonanotube, which has the dual capability of imaging with PET and MRI scans. There are apparatuses capable of performing both scans.^61^ The same research group also recorded dynamic PET/CT images on a healthy mouse with the same contrast agent, reporting its stability and quick clearance from circulation. Thus, they developed contrast agents that combine the excellent spatial and contrast resolution of MRI with the high sensitivity of PET. Also, Luo et al^62^ carried out PET/MRI dual imaging by functionalizing gadofullerenes with ^124^I to afford an ^124^I–f‐Gd_3_N@C_80_ nanoprobe. The in vivo imaging visualization with both MRI and microPET demonstrated the dual properties of the ^124^I‐f‐Gd_3_N@C_80_ agent (Figure 8).

**Figure 9:**
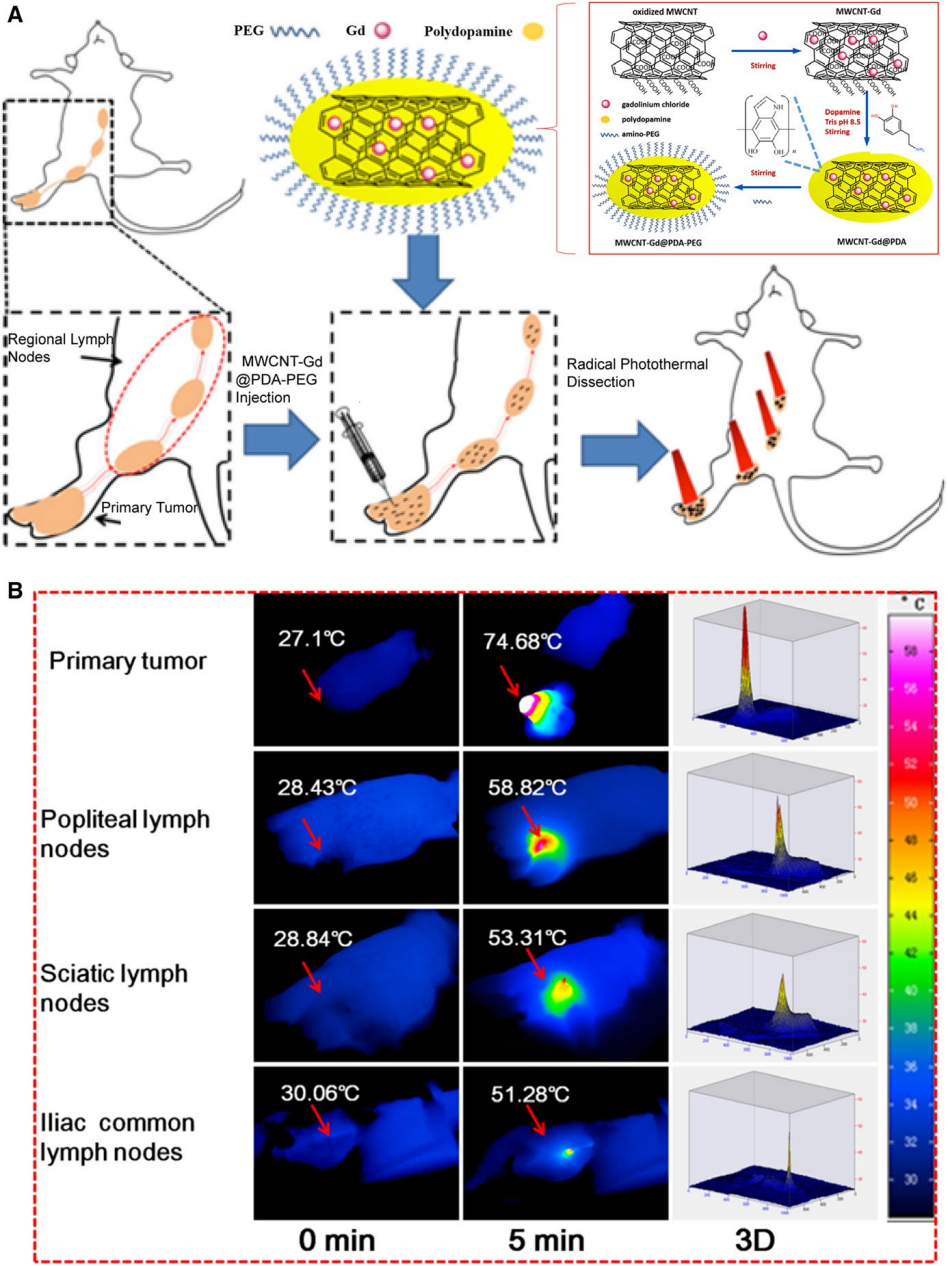
Gadonanotubes for imaging and therapy. (A) Scheme of the synthetic strategy and the T1‐MRI and colour mapping guided PTT of metastatic LNs by MWCNT‐Gd@PDA‐PEG. (B) IR thermal images of primary tumour and RLNs LNs with NIR illumination by an 808 nm laser. Reprinted with permission from,^65^ copyright 2017 Elsevier

The multifunctionality of gadonanotubes also includes the combination of imaging and therapy. Matson et al^63^ developed the conjugate ^225^Ac(III)@gadonanotubes by loading short single‐walled carbon nanotubes with both ^225^Ac(III) and Gd(III) ions. They chose the ^225^Ac(III) ion because of being a potent α‐particle (^4^He^2+^) generator with therapeutic value. Additionally, Peci et al^64^ functionalized the sidewalls of iron‐filled multi‐walled carbon nanotubes with gadolinium ions, suggesting that the complexes may be good candidates for MR imaging and magnetic hyperthermia. Recently, Wang et al^65^ fabricated a polydopamine‐encapsulated gadolinium‐loaded multi‐walled carbon nanotube (MWCNT‐Gd@DPA) for MR imaging and photothermal therapy (PPT) (Figure 9). They took advantage of the optical properties of carbon nanotubes, which have a high infrared wave range (NIR) conversion efficiency and thus can produce a great increase in local temperature that is capable of killing cancer cells. According to the in vivo and in vitro results, the conjugate was able to map the lymph nodes in mice by inducing metastasis and served a therapeutic purpose after being irradiated with a NIR laser.

More recently, Zhang et al^66^ synthesized yet another complex for imaging and therapy. They decorated multi‐walled carbon nanotubes (MWCNTs) with magneto‐fluorescent carbon quantum dots (GdN@CQDs), doxorubicin and the EGFR antibody (GP‐GdN@CQDs‐MWCNTs/DOX‐EGFR). Whereas carbon nanotubes can absorb in the near‐infrared wave range (NIR), the magnetic and fluorescent properties of the magneto‐fluorescent carbon quantum dots (GdN@CQDs) are instrumental for achieving MR and fluorescence dual‐mode imaging. Doxorubicin was selected because it is a common chemotherapy agent administered to treat several types of cancers, while the EGFR antibody has both therapeutic properties and active targeting capabilities against cancer cells. The nanocomposite was used for imaging and chemo‐photothermal therapy in nude mice bearing tumours, resulting in the elimination of 100% of the tumours.^66^

All these works have shown gadolinium‐based contrast agents have the possibility of multifunctionality overcoming the limitations of current contrast agents for multifunctional imaging and the combination of imaging and therapy.

### 2.4 Cell labelling capability

MRI is an ideal tool for in vivo imaging and tracking of engrafted cells for regenerative medicine because can image deep inside tissue, gives accurate anatomical and physiological information and offer the possibility of monitoring transplanted cells over long periods of time.^67^ However, due to their intrinsic low relaxivity and short blood circulation time, conventional gadolinium contrast agents are not suitable for cell labelling. In contrast, a great staining efficiency has been observed with gadolinium‐containing carbon nanomaterials. Anderson et al^30^ examined cell staining with polyhydroxyl Gd@C_82_ as a T1 contrast agent and found that complexing gadofullerenes with protamine sulphate (a transfection agent) increased cell labelling, allowing for visualization of the cells by MR imaging in vitro and in vivo. Rammohan et al^29^ employed gadonanodiamonds for the labelling of cancer cells (MDA‐MB‐231m‐Cherry human triple‐negative breast cancer cell line). Based in gadolinium quantification they found gadonanodiamonds confers 300‐fold improvement in cellular delivery of Gd(III) compared to Gd(III)−DOTA and conventional contrast agents (Gd–C5−COOH). Also, the MR images of a mouse bearing a gadonanodiamonds‐labelled xenograft confirmed the viability of gadonanodiamonds for MRI and cell tracking.

**Figure 10:**
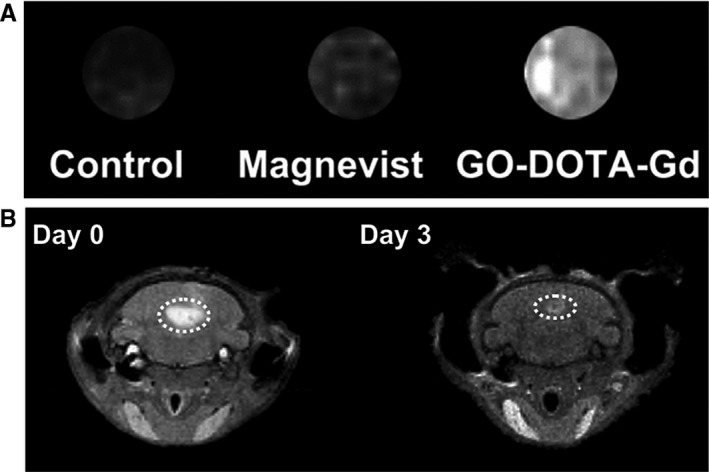
Cell labelling with gadofullerenes. (A) T1‐weighted MR images of human mesenchymal stem cells labelled with Magnevist or gadographene (GO‐DOTA‐Gd) at the same Gd concentration (25 μmol/L). The unlabelled human mesenchymal stem cells were used as the control. (B) T1‐weighted MR images of nude mice obtained on (left) day 0 and (right) day 3 after injection of gadographene (GO‐DOTA‐Gd) labelled human mesenchymal stem cells. Reprinted with permission from,^31^ copyright 2018 Elsevier

Recently, Zhang et al^31^ developed a gadographene for staining human mesenchymal stem cells. The contrast was synthesized by PEGylation of ultrasmall graphene oxide, followed by conjugation with a chelating agent (DOTA) and then Gd(III) to form GO‐DOTA‐Gd complexes. The authors demonstrated the efficient labelling of human mesenchymal stem cells by GO‐DOTA‐Gd (Figure 10A), without any apparent adverse effects on proliferation and differentiation of the cells. The cells could be visualized by MRI after implantation into nude mice in vivo (Figure 10B).

The excellent cell penetration of cell membranes by gadolinium‐loaded carbon nanomaterials and their retention inside cells may explain their cell staining capability. Some studies have evaluated the internalization and subcellular localization of gadolinium‐containing carbon nanomaterials. For instance, Sitharaman et al^68^ tested the cellular uptake and internalization of Gd@C_60_[C(COOH)_2_]_10_ in NIH 3T3 mouse fibroblast cells and bone marrow stromal cells (human mesenchymal stem cells). In both cases, they observed a saturation uptake of 0.5 mmol/L of gadolinium. Given that they did not detect any release of gadolinium from the cells within a 48‐hour period, the internalization seems to be an irreversible process.

**Figure 11:**
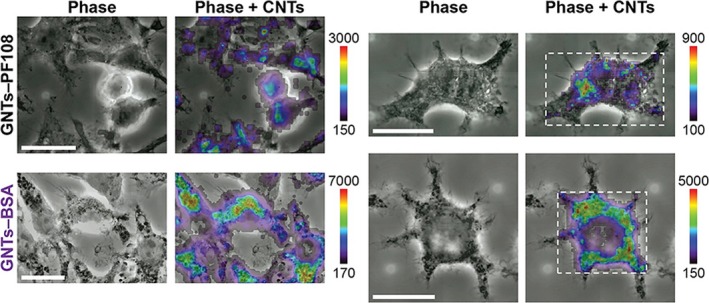
Cytoplasmatic distribution of gadofullerenes. Raman mapping of the subcellular distribution of gadonanotubes within the J774A.1 macrophage cell line. Gadonanotubes dispersed with the surfactant PF108 (GNTs—PF108) and those dispersed with BSA (GNTs‐BSA). Scale bars represent 20 μm, and the colour bar indicates the local CNT concentration. Reprinted with permission from ref,^70^ copyright 2015 American Chemical Society

The cell internalization of gadonanotubes has also been described. For instance, according to Hassan et al^69^ the internalization is less efficient for gadonanotubes dispersed with BSA than those functionalized with serine. In contrast, when working with the HELA and J774A.1 macrophage cell line, Holt et al^70^ found that gadonanotubes dispersed with BSA were more internalized than those dispersed with the PF108 surfactant. Regarding the subcellular internalization, Avti et al^71^ and Marangon et al^72^ observed that gadonanotubes tended to be compartmentalized within endosomes in the cytoplasm of NIH/3T3 cells and RAW 264.7 mouse macrophages, suggesting an active process of cellular internalization. Nevertheless, Hassan et al,^69^ Tran et al^73^ and Gizzatov et al^74^ reported the formation of gadonanotube aggregates in the cytoplasm, but without encapsulation in vesicles (endosomes). Recently, Holt et al^70^ used resonant Raman imaging to determine the subcellular distribution of gadonanotubes, finding them predominantly in a perinuclear location of the cytoplasm (Figure 11) and that dispersal agents affected the degree of agglomeration. For instance, gadonanotubes dispersed with the PF108 surfactant accumulated randomly at high concentrations in distinct regions, while, gadonanotubes dispersed with BSA were more uniformly distributed throughout the cytoplasm.

## 3 IN VITRO AND ANIMAL MODEL SAFETY ASSESSMENTS

The advantages of gadolinium‐containing carbon nanomaterials over conventional contrast agents should certainly have an impact on several areas of medicine, especially imaging and therapy for cancer patients. However, it is necessary to assure the safety of nanoparticles (NPs) interacting with the organism at the cellular and systemic level. Hence, the cytotoxicity and biodistribution of gadolinium‐containing carbon nanomaterials are presently explored to provide insights into their effects on biological systems.

### 3.1 Cytotoxicity

One of the main concerns with the medical applications of carbon nanomaterials containing gadolinium ions is their cytotoxicity, because of the well‐documented cell toxicity of carbon nanomaterials and the toxicity of Gd(III) ions. Several studies in cell cultures have shed light on cytotoxicity, cell viability and cell proliferation. Chen et al^75^ tested the cytotoxicity and cell viability of hydroxylated gadofullerenes ([Gd@C_82_(OH)_22_]n) on human hepatoma cells (HepG2) and rat hepatoma cells (RH 35), finding no toxic effects at concentrations of 2 × 10^−8^ to 1 × 10^−5^ mol/L of Gd(III). They attributed the low levels of toxicity to the hydroxyl groups, which favour the dispersion of these fullerenes. Anderson et al^30^ also analysed the cytotoxicity and cell viability of the hydroxylated gadofullerenes (Gd@C_82_(OH)_40_) in HeLa cells, human macrophages and human mesenchymal stem cells, reporting no difference with the control groups. Liu et al^76^ applied Gd@C_82_(OH)_22_ to LLC cells, B cells, T cells and macrophages, finding no significant effects in relation to cytotoxicity or cell viability with doses of Gd(III) from 0.1 µmol/L to 100 µmol/L. Sitharaman et al^68^ examined the cytotoxicity of Gd@C_60_[C(COOH)_2_]_10_ in NIH 3T3 mouse fibroblast and bone marrow stromal cells (human mesenchymal stem cells). At a concentration of 500 µmol/L of Gd(III), the cells were unaffected during a 24 hours period.

Regarding the cytotoxicity of gadonanotubes, Tang et al^77^ reported no significant effect on murine macrophage cells (J774A.1) at concentrations ≤27.75 µmol/L of Gd(III), but cytotoxicity was indeed present at higher doses >27.75 µmol/L of Gd(III). Holt et al^70^ observed no cytotoxicity on HeLa cells and murine macrophage‐like cells (J774A.1) exposed to carbon gadonanotubes at concentrations ≤27 μg/mL of carbon nanotubes. Also, Servant et al^78^ observe no significant decrease in cell viability of HUVEC cells after 4 and 24 hours of exposure to long oxidized carbon gadonanotubes at concentrations ≤40 μg mL^−1^.

In contrast to gadofullerenes, the tested gadonanotubes caused cytotoxicity at reduced concentrations >27 µmol/L of Gd(III), gadofullerenes no exhibited toxicity even at concentrations up to 500 µmol/L of Gd(III). The difference in toxicity was expected, carbon nanotubes have a fibre‐like shape and their toxic properties may be comparable to the observed with other fibrous particles.^79^ Also, the toxicity of gadonanotubes varied among different cell lines, some cells lines were more susceptible to gadonanotubes, for example The J774A.1 cell line was highly susceptible to gadonanotubes, in contrast, the NIH/3T3 cell line revealed no toxic effects to the same nanotubes at concentrations up to 100 μg/mL.^70^, ^71^, ^77^ Regarding the chemistry properties of gadonanotubes, Wang et al^65^ reported that at the same concentration (50 μg/mL) naked gadonanotubes were more cytotoxic than gadonanotubes with a shell of PDA‐PEG (polydopamine‐polyethylene glycol) in the BxPC‐3 cell line. This result agrees with previous reports that found the functionalization of carbon nanotubes can reduce their toxicity.^80^

Recently, the cytotoxicity of gadonanodiamonds was reported. Rammohan et al^29^ demonstrated gadonanodiamonds at concentrations up to 1000 μg/mL were well tolerated by the MDA‐MB‐231 cell line. Also, they found the metabolic activity of the SKOV‐3, HeLa and NIH/3T3 cell lines was not affected by the exposition to gadonanodiamonds.

Although most studies shown a low grade of toxicity at the tested doses, there is a necessity to understand the interaction between gadolinium‐containing carbon nanomaterials and cells for the rational design of contrast agents with biocompatibility and multifunctionality. Considering the discussed studies, it is recommendable the addition of control groups for future research studies (eg for carbon nanomaterials and gadolinium ions). They may help to elucidate the toxicity of each component and the toxicity related to the complex. Also, due to the production of mono‐disperse contrasts with homogeneous gadolinium load is still in its early stages, the robust characterization of the synthesized contrasts may help to establish a proper relationship between the properties of the nanomaterials and their toxicity.

### 3.2 In vivo toxicity and biodistribution

In vivo studies are indispensable for determining the efficacy and toxicity of gadolinium‐containing carbon nanomaterials. They may help to determine how these new contrast agents are biodistributed, what is the clearance mechanisms, the toxicity related with the materials, the maximum tolerable doses, and the assessment of important parameters that may help to determine whether the contrasts are safety for clinical applicability. Table 1 summary the major biological findings from in vivo studies of gadofullerenes and gadonanotubes.

**Table 1:** Biodistribution studies of gadolinium‐containing carbon nanomaterials as contrast agents (in chronological order)

Nanomaterial	Animal model	Dose administered	Biological response	Ref.
Gd@C_82_(OH)_40_	CDF1 mice	5 μmol Gd/kg	Gd@C_82_(OH)_40_ found in the reticular‐endothelial system	12
Gd@C_82_(OH)_40_	CDF1 mice	5 μmol Gd/kg	Gd‐fullerenols observed in the reticular‐endothelial system	44
Gd@C_60_[C(COOH)_2_]_10_	Fischer 344 female rats	35 mg/kg	Reticular‐endothelial system with polyhydroxyl fullerene	45
Gd@C_82_(OH)_22_	Kunming mice (tumour model)	114 and 228 µg/kg and 10‐7 mol/kg	Antineoplastic activity	75
Gd_3_N@C_80_[DiPEG5000‐(OH)_x_]	Fischer 344 female rats (glioma tumour Model)	0.0131 mmol/L	No toxic effect	51
Gd@C_82_(OH)_40_	Sprague‐Dawley rats	Human mesenchymal stem cells stained with 1.19 × 10^−10^ mg per cell	No toxic effect	30
Gd@C_82_(OH)_22_	C57BL/6 mice (tumour model)	0.1 or 0.5 mmol/kg	Immune system response	76
Gd_3_N@C_80_(OH)_∼26_(CH_2_CH_2_COO*_m_*)_∼16_	Fischer 344T9 rats with brain tumours	0.0475 mmol/L	Prolonged presence of contrast at 7 d post‐injection	98
Gd_3_N@C_80_[DiPEG350 (OH)*x*]	Brain tumour model	0.0235 mmol/L	Nanoparticles remained after 7 d	99
Gd_3_N@C_80_(IL‐13 peptide)	Athymic Nu/Un, brain tumour model	0.2 μL/mL	Preferential accumulation in tumours	27
^177^Lu‐DOTA‐f‐Gd_3_N@C_80_	Brain tumour model	1.11 MBq of 177 Lu‐DOTA‐f‐Gd_3_N@C _80_	Nearly 37.5% of the complex remained intact after 52 d	89
Gd@C_82_(OH)_22_	BALB/c mice	2 μmol/kg (once a day for 6 times)	Inflammatory response	81
MWNT/GdL GdL = Gd(III) + DTPA	BALB/c mice	50 µL of 0.05 mmol/L GdL	100% survival of animals after over 1 mo	100
Gd‐SWCNTs	Wistar rats	0.5 mg/kg	No inflammation or organ damage, but a differential effect at the genomic level	82
Gd‐CNTs Gd = Gd(III) + DTPA	C57/Bl6 mice	5.75 μmol/kg	Signal enhancement in the liver and spleen, and to a much greater extent in the bladder	72
^64^Cu@GNTs	Normal, tumour‐free athymic nude mice	7.4‐11.1 MBq of surfactant‐wrapped ^64^Cu@GNTs	Greater accumulation in the lungs	60
IL‐13‐Gd_3_N@C_80_(OH)_x_(NH_2_)_y_	Brain tumour model	~250 µL (~0.25 nmol)	Preferential accumulation in tumours	23
Gd@C_82_(O)_10_(OH)_22_ Gd@C_82_(O)_10_(OH)_16_	BALB/c mice	48 μmol/kg	Nanoparticles accumulated in the reticular‐endothelial system	90
NDs‐Gd Gd = Gd(III) + DOTA	SCID‐beige mice	Cells labelled with NDG at a concentration of 500 μmol/L Gd(III)	Preferential accumulation in tumours	29
ZD2‐Gd_3_N@C_80_	Tumour model	1.7 μmol/kg^−1^	Dense accumulation in tumours	25
MWCNT‐Gd@PDA‐PEG	Male nude mice	50 mL of 3 mg/mL	Preferential accumulation in tumours and in regional lymph nodes	65
PEG‐GMF‐PPy NP	Tumour model	100 μL (Gd concentration: 50 ppm) of PEG‐GMF‐PPy	Preferential accumulation in tumours and no noticeable organ damage	101

Until now, most of the revised studies were focused to prove the potential application of the contrast agents and complementary efforts were done to understand their toxicity. As can be appreciated in Table 1, most of the studies reported no acute effects, and just three studies described gadonanotubes and gadofullerenes may promote inflammation and effects at genomic level.^76^, ^81^, ^82^ However, due to the well‐known toxicity of carbon nanomaterials and the toxicity of free gadolinium ions, more studies are needed to clarify the clinical application. For instance, previous works reported carbon nanotubes and fullerenes may promote adverse effects, including inflammation, oxidative stress, DNA damage and mutation, malignant transformation, the formation of granulomas and interstitial fibrosis.^79^, ^83^, ^84^ Also, owing the toxicity of free gadolinium ions, it is essential to carry out bioavailability studies by their potential dissociation from the carbon nanostructure. It takes significance because even metal impurities can be released from the carbon nanotube structure despite their apparent encapsulation by carbon.^85^ Particularly, the concern emerges by the presence of gadolinium in the brain of patients that received repeated administration of chelated gadolinium contrast agents for MRI.^10^, ^86^ The dechelation of free Gd(III) ions from unstable contrast agents is considered the main cause for the observed accumulation.^87^ Also, it was reported a relation between the onset of nephrogenic systemic fibrosis (NSF) and the administration of gadolinium contrast agents in patients suffering from severe renal disease.^10^ Although the pathogenic mechanism of nephrogenic systemic fibrosis is not fully understood, the presence of free Gd(III) ions is considered as a possible cause. In patients with normal renal function, gadolinium contrasts have a pharmacokinetic half‐life on the order of 1‐2 hours and are excreted unchanged predominantly through the kidneys. However, for patients with renal failure, the pharmacokinetic half‐life increases due to the low elimination rate, and in that way, the risk of enzymatic dechelation can increase and thus the presence of toxic gadolinium ions.^10^

Several studies have tested the stability of the new contrasts, for instance, Sitharaman et al^13^ used triethylenetetranitrilohexaacetic acid (TTHA), a compound with high denticity that fills all the co‐ordination sites of Gd(III) ions, thus preventing the formation of an inner sphere and inhibiting any improvement in water relaxivity. When they examined the relaxivity of their gadonanotubes with and without TTHA, there were no differences between the groups, corroborating the absence of free Gd(III) ions. The stability of gadonanotubes has also been tested assessing the presence of free Gd(III) in the supernatant after the proper washing of gadonanotubes, by inductively coupled plasma optical emission spectroscopy (ICP‐OES) the supernatants showed no evidence of free ions.^76^ Regarding gadofullerenes, Gd(III) ions are encapsulated in the fullerene cage, an extraordinarily stable structure that is extremely unlikely to break and release the toxic ions. Han et al^25^ discarded the possible release of free Gd(III) ions in vitro by performing a transmetalation assay, finding gadofullerenes functionalized with the short peptide (ZD2) to be stable under biological conditions (eg human serum). Despite the above results, there is a necessity to study the stability in physiological conditions over long periods of time, most of the previous reports analysed the stability during a short time.

**Figure 12:**
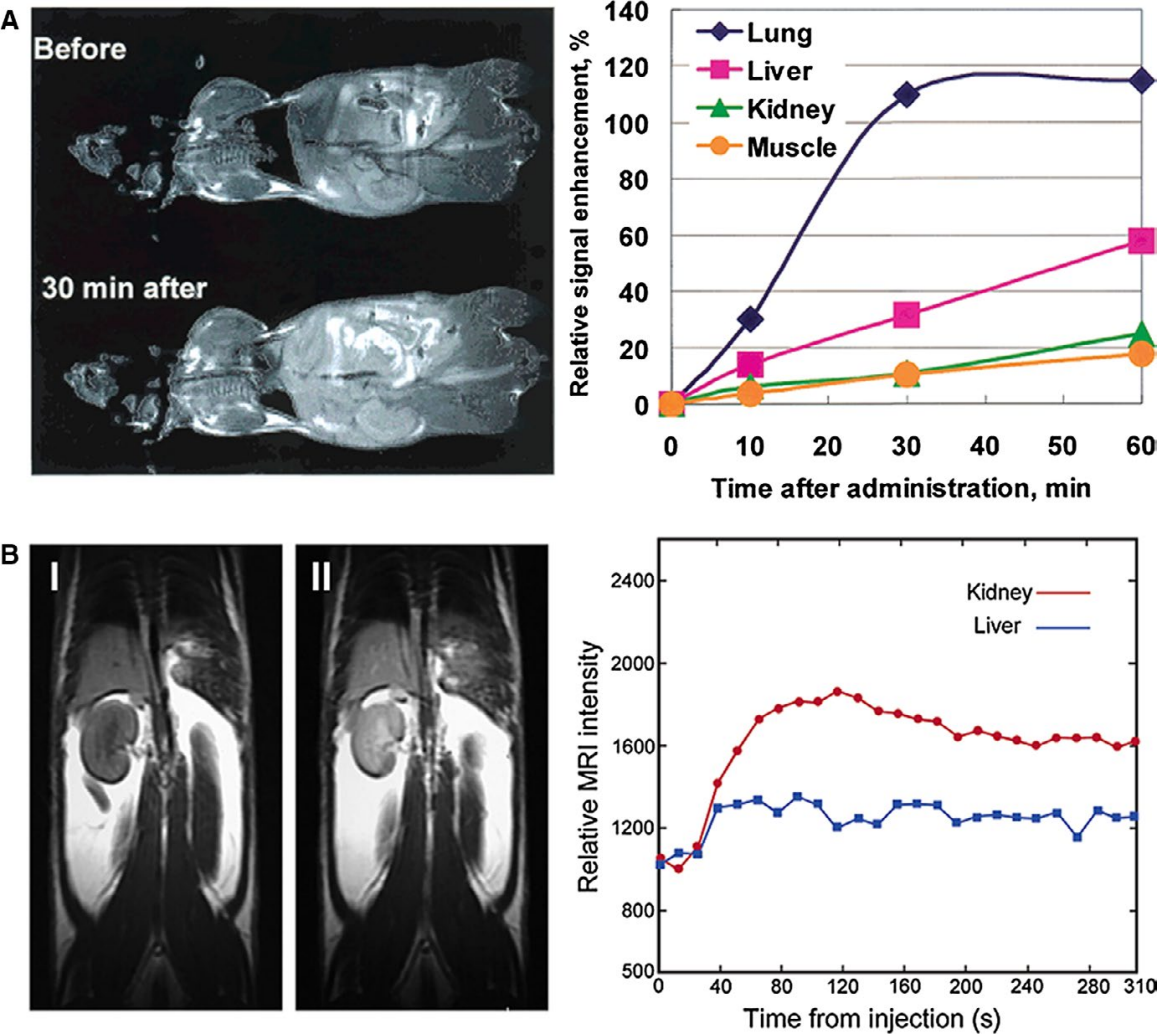
Biodistribution studies of gadofullerenes. (A) T1‐weighted MRI of CDF1 mice before and 30 min after intravenous administration of Gd@C_82_(OH)_40_ via the tail vein (left) and its time‐dependent signal intensity changes in various organs (right). Reprinted with permission from,^12^ copyright 2001 American Chemical Society. (B) T1‐weighted MRI of CDF1 mice before and 16 min after administration of Gd@C_60_[C(COOH)_2_]_10_. Increased signal intensity is displayed in the kidney (left); representative in vivo MRI intensity‐derived biodistribution data showing the Gd@C_60_[C(COOH)_2_]_10_ signal enhancement within the first 5 min of administration, revealing rapid renal uptake with a minimum of liver uptake (red circles, kidney; blue squares, liver) (right). Reprinted with permission from,^45^ copyright 2003 American Chemical Society

In terms of biodistribution, most of the gadolinium‐containing carbon nanomaterials tend to be entrapped by the mononuclear phagocyte system, a network of immune and architectural cells that removes foreign material from the bloodstream.^88^ The system encompasses monocytes of the blood, macrophages in connective tissue, lymphoid organs, bone marrow, bone, liver and lung.^88^ For instance, Mikawa et al^12^ evaluated the biodistribution of the hydroxylated gadofullerenes (Gd@C_82_(OH)_40_) in CDF1 mice. Testing the concentration of gadolinium in different organs by ICP‐AES (Inductively coupled plasma atomic emission spectroscopy) they found that after their intravenous injection the gadofullerenes were mainly accumulated in lung, liver and spleen (Figure 12A) and 24 hours later they observed a clearance from lungs, meanwhile the concentration in liver and spleen was constant. In a subsequent study, they identified the same biodistribution pattern for a sample of Gd@C_82_(OH)_40_ of high purity.^44^ Then, Bolskar et al^17^ reported the Gd@C_60_[C(COOH)_2_]_10_ was mainly accumulated in kidneys and by the presence of gadofullerenes in urine they inferred the gadofullerenes could be eliminated via renal filtration. Chen et al^81^ found Gd@C_82_(OH)_22_ was mainly distributed in bone followed by pancreas, spleen, kidney and liver. Also, they reported that 24 hours after intraperitoneal administration 50% of the gadofullerenes were excreted by urine and 35% by faeces.^66^ Shultz et al^89^ reported the f‐Gd_3_N@C_80_ was mainly accumulated in liver, lung, brain and spleen after brain infusion administration. Then, after 7 days from initial administration they reported a clearance from liver, lungs and spleen, however, the presence of a residual signal of gadofullerenes was observed in all the organs. Han et al^25^ reported the ZD2‐Gd_3_N@C80 had a significant preferential accumulation in kidneys and bladder, and then, after 1 week most of the gadofullerenes were eliminated via renal clearance. Recently, Li et al,^90^ analysed the biodistribution of Gd@C_82_(O)_10_(OH)_22_ and Gd@C_82_(O)_10_(OH)_16_ and according with T1 MR images the gadofullerenes were mainly accumulated in liver.

The biodistribution of gadonanotubes has also been reported. Avti et al^82^ found synthesized gadonanotubes (average diameter 2.05 nm, length 500 nm to 1.5 mm) using gadolinium nanoparticles as catalysts were mainly accumulated in lungs, brain, kidney, liver and spleen, and based on the biodistribution profile they suggested the gadonanotubes were cleared by renal filtration and hepatobiliary excretion. Marangon et al^72^ evaluated the biodistribution of functionalized gadonanotubes (diameter of 20‐30 nm, average length of ≈400 nm) at 12.5 mg/kg and found a high accumulation in bladder after initial administration, then after 5.3 hours they observed a significant decline from bladder. The rapid clearance suggested most of the gadonanotubes were eliminated through the renal excretion route. Also, they found the gadonanotubes induce neither tissue damage, nor inflammatory response, and they do not promote cellular toxicity. Cisneros et al^60^ reported a high lung uptake of surfactant‐wrapped 64Cu@GNTs (average diameter 1.4 nm, length 20 nm to 80 nm) and a high retention of the complex, even after 48 hours. Whang et al^65^ investigated the biodistribution of GdN@CQDs‐MWCNTs (mean diameter of 30 nm with length of about 100‐300 nm) and reported a high accumulation in liver 4 hours after intravenous injection. They analysed the potential adverse side effects by evaluating the body weight of mice, which was found to be stable.

As can be appreciated, most of the gadolinium‐containing carbon nanomaterials tend to be entrapped by filtration organs (eg liver, lungs, bladder, spleen and kidneys). The patter agrees with those reported for fullerenes and carbon nanotubes. For example, it was reported that long carbon nanotubes are mainly accumulated in lungs,^91^ Avti et al^82^ observed the same patter for gadonanotubes of 500 nm to 1.5 mm of length, by contrast. Also, it is well‐known small carbon nanotubes can be accumulated in bladder and kidneys,^91^ Marangon et al^72^ observed the functionalized gadonanotubes with average length of ≈400 nm, were mainly accumulated in bladder and kidneys.

Until now, most of the reviewed works reported elimination percentages of gadonanotubes and gadofullerenes, but not the complete elimination from organs. This long‐term accumulation may represent a risk. A number of reports have found pristine CNTs remain in lungs for months or even years after pulmonary deposition, even more, the accumulation is associated with chronic inflammation and tissue damage.^84^, ^91^ Particularly, the accumulation of MWCNTs in lungs is associated with fibrotic‐like lesions via non‐resolved chronic inflammation^83^ and the chronic exposure to SWCNTs may cause malignant transformation of human lung epithelial cells and induced tumorigenesis in mice.^92^ The hepatic accumulation of carbon nanotubes is associated with the induction of a local immune response and liver damage.^93^ The accumulation in kidney may induce toxicity due increased oxidative stress, mitochondrial damage or DNA damage.^94^, ^95^ In spleen, the accumulation of carbon nanotubes has been associated with immunotoxicity.^96^

As can be appreciated, carbon nanotubes and fullerenes are associated with toxic effects, however, also a number of reports have found the functionalization with functional groups, polymers and biomolecules may modulate the biodistribution and elimination.^32^, ^91^ Carboxylic acid‐functionalized fullerenes have exhibited high levels of retention (even 72 hours after intravenous administration) in contrast hydroxyl‐functionalized fullerenes are rapidly excreted in urine.^97^ The functionalization also improves the dispersion of carbon nanomaterials, Marangon et al^72^ found well‐dispersed carbon nanotubes prevent lung accumulation and can be eliminated in urine. It is also important to remark the preferred accumulation in a desired tissue can be modulated by the functionalization of the carbon structure with antibodies, peptides or polysaccharides. Fillmore et al,^27^ Zheng et al^55^ and Han et al^25^ reported the active targeting of implanted tumours in mice functionalizing gadofullerenes with peptides with high affinity by overexpressed membrane proteins on tumour cells or extracellular matrix components.

As shown, new formulations of gadolinium‐containing carbon nanomaterials are being evaluated in laboratory models. However, more in vitro and in vivo studies are essential to ensure the new contrast agents are useful for MR imaging. Much remains unknown, it is recommended future research evaluate the chronic toxicity over long periods of time and the acute toxicity administrating high doses, the transport mechanisms, fate and adverse health effects due gadolinium‐containing carbon nanomaterials.

## 4 CONCLUSION

A critical evaluation was conducted of the advantages of gadolinium‐containing carbon nanomaterials over conventional chelated gadolinium contrast agents. Their enhanced relaxivity allows for reliable imaging of cellular and molecular biomarkers with a lower dose of Gd(III), thus reducing the risk of toxicity from free Gd(III) ions. The physical and chemical properties of carbon nanomaterials have enabled the fabrication of contrast agents with multifunctionality as well as with active targeting capability, which encompasses multimodal imaging and the combination of imaging with therapy with high specificity. Nowadays, there are intense efforts to design new T1 MRI nanoprobes based on gadolinium‐containing carbon nanomaterials. However, several issues should be overcome, the production of mono‐disperse nanomaterials with controlled size and a homogeneous gadolinium load is still in its early stages, and require better and standardized synthesis methods. The new contrast must be tested with standard characterization techniques and valid methods to establish the proper relationship between the characteristics of the nanomaterial and its probable toxicity in order to understand its behaviour in living systems. Also, more preclinical studies as biodistribution, biocompatibility, pharmacokinetics and long‐term stability are needed to clarify the potential clinical use of these nanomaterials.

## CONFLICT OF INTEREST

No conflict of interest exits in the submission of this manuscript.

## AUTHOR CONTRIBUTION

ARG performed the literature research and drafted the manuscript; MR, PGL, LAM and VAB revised the manuscript, drafted the manuscript, edited the final version and gave the final approval for the article to be published.

## Data Availability

Data sharing is not applicable to this article.

## References

[1] Weller M , van den Bent M , Hopkins K , et al. EANO guideline for the diagnosis and treatment of anaplastic gliomas and glioblastoma. Lancet Oncol. 2014;15:e395‐e403. 25079102 10.1016/S1470-2045(14)70011-7

[2] Tempany CMC , Jayender J , Kapur T , et al. Multimodal imaging for improved diagnosis and treatment of cancers. Cancer. 2015;121:817‐827. 25204551 10.1002/cncr.29012 PMC4352132

[3] Constantine G , Shan K , Flamm SD , Sivananthan MU . Role of MRI in clinical cardiology. Lancet. 2004;363:2162‐2171. 15220041 10.1016/S0140-6736(04)16509-4

[4] Nitz WR , Reimer P . Contrast mechanisms in MR imaging. Eur Radiol. 1999;9:1032‐1046. 10415233 10.1007/s003300050789

[5] Trattnig S , Pinker K , Ba‐Ssalamah A , Nöbauer‐Huhmann I‐M . The optimal use of contrast agents at high field MRI. Eur Radiol. 2006;16:1280‐1287. 16508769 10.1007/s00330-006-0154-0

[6] U.S. Food and Drug Administration . Developing medical imaging drug and biological products part 1: Conducting safety assessments; 2019.

[7] Ni D , Bu W , Ehlerding EB , Cai W , Shi J . Engineering of inorganic nanoparticles as magnetic resonance imaging contrast agents. Chem Soc Rev. 2017;46:7438‐7468. 29071327 10.1039/c7cs00316a PMC5705441

[8] Helm L . Optimization of gadolinium‐based MRI contrast agents for high magnetic‐field applications. Future Med Chem. 2010;2:385‐396. 21426173 10.4155/fmc.09.174

[9] Peng Y‐K , Tsang SCE , Chou P‐T . Chemical design of nanoprobes for T1‐weighted magnetic resonance imaging. Mater Today. 2016;19:336‐348.

[10] Kanal E , Tweedle MF . Residual or retained gadolinium: practical implications for radiologists and our patients. Radiology. 2015;275:630‐634. 25942418 10.1148/radiol.2015150805

[11] Ananta JS , Godin B , Sethi R , et al. Geometrical confinement of gadolinium‐based contrast agents in nanoporous particles enhances T1 contrast. Nat Nanotechnol. 2010;5:815‐821. 20972435 10.1038/nnano.2010.203 PMC2974055

[12] Mikawa M , Kato H , Okumura M , et al. Paramagnetic water‐soluble metallofullerenes having the highest relaxivity for MRI contrast agents. Bioconjugate Chem. 2001;12:510‐514. 10.1021/bc000136m 11459454

[13] Sitharaman B , Kissell KR , Hartman KB , et al. Superparamagnetic gadonanotubes are high‐performance MRI contrast agents. Chem Commun. 2005;915‐917. 10.1039/b504435a 16075070

[14] Sitharaman B , Wilson LJ . Gadonanotubes as new high‐performance MRI contrast agents. Int J Nanomed. 2006;1:291‐295. PMC2426797 17717970

[15] Sethi R , Mackeyev Y , Wilson LJ . The Gadonanotubes revisited: a new frontier in MRI contrast agent design. Inorg Chim Acta. 2012;393:165‐172.

[16] Kuźnik N , Tomczyk MM . Multiwalled carbon nanotube hybrids as MRI contrast agents. Beilstein J Nanotechnol. 2016;7:1086‐1103. 27547627 10.3762/bjnano.7.102 PMC4979685

[17] Bolskar RD . Gadofullerene MRI contrast agents. Nanomedicine. 2008;3:201‐213. 18373426 10.2217/17435889.3.2.201

[18] Zheng J‐P , Zhen M‐M , Wang C‐R , Shu C‐Y . Recent progress of molecular imaging probes based on gadofullerenes. Chinese J Anal Chem Chinese. 2012;40:1607‐1615.

[19] Popov AA , Yang S , Dunsch L . Endohedral fullerenes. Chem Rev. 2013;113:5989‐6113. 23635015 10.1021/cr300297r

[20] Ghiassi KB , Olmstead MM , Balch AL . Gadolinium‐containing endohedral fullerenes: structures and function as magnetic resonance imaging (MRI) agents. Dalton Trans. 2014;43:7346‐7358. 24522668 10.1039/c3dt53517g

[21] Li T , Dorn HC . Biomedical applications of metal‐encapsulated fullerene nanoparticles. Small. 2017;13:1603152. 10.1002/smll.201603152 28026111

[22] Sitharaman B , Wilson LJ . Gadofullerenes and gadonanotubes: a new paradigm for high‐performance magnetic resonance imaging contrast agent probes. J Biomed Nanotechnol. 2007;3:342‐352.

[23] Li T , Murphy S , Kiselev B , et al. A new interleukin‐13 amino‐coated gadolinium metallofullerene nanoparticle for targeted MRI detection of glioblastoma tumor cells. J Am Chem Soc. 2015;137:7881‐7888. 26022213 10.1021/jacs.5b03991

[24] Bianco A , Kostarelos K , Partidos CD , Prato M . Biomedical applications of functionalised carbon nanotubes. Chem Commun. 2005;571‐577. 10.1039/b410943k 15672140

[25] Han Z , Wu X , Roelle S , Chen C , Schiemann WP , Lu Z‐R . Targeted gadofullerene for sensitive magnetic resonance imaging and risk‐stratification of breast cancer. Nat Commun. 2017;8:692. 28947734 10.1038/s41467-017-00741-y PMC5612990

[26] Shu C‐Y , Ma X‐Y , Zhang J‐F , et al. Conjugation of a water‐soluble gadolinium endohedral fulleride with an antibody as a magnetic resonance imaging contrast agent. Bioconjugate Chem. 2008;19:651‐655. 10.1021/bc7002742 18254583

[27] Fillmore HL , Shultz MD , Henderson SC , et al. Conjugation of functionalized gadolinium metallofullerenes with IL‐13 peptides for targeting and imaging glial tumors. Nanomedicine. 2011;6:449‐458. 21542684 10.2217/nnm.10.134

[28] Na HB , Hyeon T . Nanostructured T1 MRI contrast agents. J Mater Chem. 2009;19:6267‐6273.

[29] Rammohan N , MacRenaris KW , Moore LK , et al. Nanodiamond–gadolinium(III) aggregates for tracking cancer growth *in vivo* at high field. Nano Lett. 2016;16:7551‐7564. 27960515 10.1021/acs.nanolett.6b03378 PMC5482002

[30] Anderson SA , Lee KK , Frank JA . Gadolinium‐fullerenol as a paramagnetic contrast agent for cellular imaging. Invest Radiol. 2006;41:332‐338. 16481917 10.1097/01.rli.0000192420.94038.9e

[31] Zhang M , Liu X , Huang J , et al. Ultrasmall graphene oxide based T1 MRI contrast agent for *in vitro* and *in vivo* labeling of human mesenchymal stem cells. Nanomedicine: NBM. 2018;14:2475‐2483. 10.1016/j.nano.2017.03.019 28552648

[32] Hong G , Diao S , Antaris AL , Dai H . Carbon nanomaterials for biological imaging and nanomedicinal therapy. Chem Rev. 2015;115:10816‐10906. 25997028 10.1021/acs.chemrev.5b00008

[33] Rodríguez‐Galván A , Amelines‐Sarria O , Rivera M , del Carreón‐Castro MP , Basiuk VA . Adsorption and self‐assembly of anticancer antibiotic doxorubicin on single‐walled carbon nanotubes. NANO. 2015;11:1650038.

[34] Rodríguez‐Galván A , Heredia A , Amelines‐Sarria O , Rivera M , Medina LA , Basiuk VA . Non‐covalent attachment of silver nanoclusters onto single‐walled carbon nanotubes with human serum albumin as linking molecule. Appl Surf Sci. 2015;331:271‐277.

[35] Zhang Z , Nair SA , McMurry TJ . Gadolinium meets medicinal chemistry: MRI contrast agent development. Curr Med Chem. 2005;12:751‐778. 15853710 10.2174/0929867053507379

[36] Zhang S , Sun D , Li X , Pei F , Liu S . Synthesis and solvent enhanced relaxation property of water‐soluble endohedral metallofullerenols. Fullerene Sci Technol. 1997;5:1635‐1643.

[37] Manus LM , Mastarone DJ , Waters EA , et al. Gd(III)‐nanodiamond conjugates for MRI contrast enhancement. Nano Lett. 2010;10:484‐489. 20038088 10.1021/nl903264h PMC2829273

[38] Shen A‐J , Li D‐L , Cai X‐J , et al. Multifunctional nanocomposite based on graphene oxide for *in vitro* hepatocarcinoma diagnosis and treatment. J Biomed Mater Res, Part A. 2012;100A:2499‐2506. 10.1002/jbm.a.34148 22623284

[39] Tóth É , Helm L , Merbach A . Relaxivity of gadolinium(III) complexes: theory and mechanism In: Merbach A, Helm L, Tóth É, eds. The Chemistry of Contrast Agents in Medical Magnetic Resonance Imaging. United Kingdom: John Wiley & Sons, Ltd; 2013:25‐81.

[40] Ma Q , Jebb M , Tweedle MF , Wilson LJ . The gadonanotubes: structural origin of their high‐performance MRI contrast agent behavior. J Mater Chem B. 2013;1:5791‐5797. 32261236 10.1039/c3tb20870b

[41] Kato H , Suenaga K , Mikawa M , et al. Syntheses and EELS characterization of water‐soluble multi‐hydroxyl Gd@C_82_ fullerenols. Chem Phys Lett. 2000;324:255‐259.

[42] Nishibori E , Iwata K , Sakata M , et al. Anomalous endohedral structure of Gd@C_82_ metallofullerenes. Phys Rev B. 2004;69:113412.

[43] Bottrill M , Kwok L , Long NJ . Lanthanides in magnetic resonance imaging. Chem Soc Rev. 2006;35:557‐571. 16729149 10.1039/b516376p

[44] Okumura M , Mikawa M , Yokawa T , Kanazawa Y , Kato H , Shinohara H . Evaluation of water‐soluble metallofullerenes as MRI contrast agents. Acad Radiol. 2002;9:S495‐S497. 12188319 10.1016/s1076-6332(03)80274-x

[45] Bolskar RD , Benedetto AF , Husebo LO , et al. First soluble M@C_60_ derivatives provide enhanced access to metallofullerenes and permit *in vivo* evaluation of Gd@C_60_[C(COOH)_2_]_10_ as a MRI contrast agent. J Am Chem Soc. 2003;125:5471‐5478. 12720461 10.1021/ja0340984

[46] Laus S , Sitharaman B , Tóth É , et al. Destroying gadofullerene aggregates by salt addition in aqueous solution of Gd@C_60_(OH)_x_ and Gd@C_60_[C(COOH_2_)]_10_ . J Am Chem Soc. 2005;127:9368‐9369. 15984854 10.1021/ja052388+ PMC2597542

[47] Tóth É , Bolskar RD , Borel A , et al. Water‐soluble gadofullerenes: toward high‐relaxivity, pH‐responsive MRI contrast agents. J Am Chem Soc. 2005;127:799‐805. 15643906 10.1021/ja044688h

[48] Moghaddam SE , Sethi R , Shayeganfar F , Bryant RG , Wilson LJ . The gadonanotubes as high‐performance MRI contrast agents: the unappreciated role of the carbon nanotube component at low magnetic fields. Ecs J Solid State Sc. 2017;6:M3173‐M3180.

[49] Sitharaman B , Bolskar RD , Rusakova I , Wilson LJ . Gd@C_60_[C(COOH)_2_]_10_ and Gd@C_60_(OH)_x_: nanoscale aggregation studies of two metallofullerene MRI contrast agents in aqueous solution. Nano Lett. 2004;4:2373‐2378.

[50] Laus S , Sitharaman B , Tóth É , et al. Understanding paramagnetic relaxation phenomena for water‐soluble gadofullerenes. J Phys Chem C. 2007;111:5633‐5639.

[51] Fatouros PP , Corwin FD , Chen Z‐J , et al. *In vitro* and *in vivo* imaging studies of a new endohedral metallofullerene nanoparticle. Radiology. 2006;240:756‐764. 16837672 10.1148/radiol.2403051341

[52] Enochs WS , Harsh G , Hochberg F , Weissleder R . Improved delineation of human brain tumors on MR images using a long‐circulating, superparamagnetic iron oxide agent. J Magn Reason Imaging. 1999;9:228‐232. 10.1002/(sici)1522-2586(199902)9:2<228::aid-jmri12>3.0.co;2-k 10077018

[53] Allen MJ , MacRenaris KW , Venkatasubramanian PN , Meade TJ . Cellular delivery of MRI contrast agents. Chem Biol. 2004;11:301‐307. 15123259 10.1016/j.chembiol.2004.03.003

[54] Rosenblum D , Joshi N , Tao W , Karp JM , Peer D . Progress and challenges towards targeted delivery of cancer therapeutics. Nat Comm. 2018;9:1410. 10.1038/s41467-018-03705-y PMC5897557 29650952

[55] Zheng J , Liu Q , Zhen M , et al. Multifunctional imaging probe based on gadofulleride nanoplatform. Nanoscale. 2012;4:3669‐3672. 22617872 10.1039/c2nr30836c

[56] Gu M‐J , Li K‐F , Zhang L‐X , et al. *In vitro* study of novel gadolinium‐loaded liposomes guided by GBI‐10 aptamer for promising tumor targeting and tumor diagnosis by magnetic resonance imaging. Int J Nanomed. 2015;10:5187‐5204. 10.2147/IJN.S84351 PMC4544817 26316749

[57] Zhang L‐X , Li K‐F , Wang H , et al. Preparation and *in vitro* evaluation of a MRI contrast agent based on aptamer‐modified gadolinium‐loaded liposomes for tumor targeting. AAPS PharmSciTech. 2017;18:1564‐1571. 27604884 10.1208/s12249-016-0600-5

[58] Rodríguez‐Galván A , Contreras‐Torres FF , Basiuk EV , Alvarez‐Zauco E , Heredia A , Basiuk VA . Aggregation of human serum albumin on graphite and single‐walled carbon nanotubes as studied by scanning probe microscopies. J Nanosci Nanotechno. 2011;11:5491‐5498. 10.1166/jnn.2011.3435 21770209

[59] Rodríguez‐Galván A , Contreras‐Torres FF , Basiuk EV , Heredia A , Basiuk VA . Deposition of silver nanoparticles onto human serum albumin‐functionalised multi‐walled carbon nanotubes. Can J Chem Eng. 2013;91:264‐270.

[60] Cisneros BT , Law JJ , Matson ML , Azhdarinia A , Sevick‐Muraca EM , Wilson LJ . Stable confinement of positron emission tomography and magnetic resonance agents within carbon nanotubes for bimodal imaging. Nanomedicine. 2014;9:2499‐2509. 24628687 10.2217/nnm.14.26 PMC4167560

[61] Liu Z , Cai W , He L , et al. *In vivo* biodistribution and highly efficient tumour targeting of carbon nanotubes in mice. Nat Nanotechnol. 2007;2:47‐52. 18654207 10.1038/nnano.2006.170

[62] Luo J , Wilson JD , Zhang J , et al. A dual PET/MR imaging nanoprobe: ^124^I labeled Gd_3_N@C_80_ . Appl Sci‐Basel. 2012;2:465‐478.

[63] Matson ML , Villa CH , Ananta JS , Law JJ , Scheinberg DA , Wilson LJ . Encapsulation of α‐particle–emitting ^225^Ac^3+^ ions within carbon nanotubes. J Nucl Med. 2015;56:897‐900. 25931476 10.2967/jnumed.115.158311 PMC4863440

[64] Peci T , Dennis TJS , Baxendale M . Iron‐filled multiwalled carbon nanotubes surface‐functionalized with paramagnetic Gd(III): a candidate dual‐functioning MRI contrast agent and magnetic hyperthermia structure. Carbon. 2015;87:226‐232.

[65] Wang S , Lin Q , Chen J , Gao H , Fu D , Shen S . Biocompatible polydopamine‐encapsulated gadolinium‐loaded carbon nanotubes for MRI and color mapping guided photothermal dissection of tumor metastasis. Carbon. 2017;112:53‐62.

[66] Zhang M , Wang W , Wu F , Yuan P , Chi C , Zhou N . Magnetic and fluorescent carbon nanotubes for dual modal imaging and photothermal and chemo‐therapy of cancer cells in living mice. Carbon. 2017;123:70‐83.

[67] Srivastava AK , Kadayakkara DK , Bar‐Shir A , Gilad AA , McMahon MT , Bulte JWM . Advances in using MRI probes and sensors for *in vivo* cell tracking as applied to regenerative medicine. Dis Model Mech. 2015;8:323‐336. 26035841 10.1242/dmm.018499 PMC4381332

[68] Sitharaman B , Tran LA , Pham QP , et al. Gadofullerenes as nanoscale magnetic labels for cellular MRI. Contrast Media Mol. 2007;2(3):139‐146. 10.1002/cmmi.140 17583898

[69] Hassan AA , Chan BT‐Y , Tran LA , et al. Serine‐derivatized gadonanotubes as magnetic nanoprobes for intracellular labeling. Contrast Media Mol. 2010;5(5):34‐38. 10.1002/cmmi.293 20101755

[70] Holt BD , Law JJ , Boyer PD , Wilson LJ , Dahl KN , Islam MF . Subcellular partitioning and analysis of Gd^3+^‐loaded ultrashort single‐walled carbon nanotubes. ACS Appl Mater Interfaces. 2015;7:14593‐14602. 26098461 10.1021/acsami.5b04851

[71] Avti PK , Caparelli ED , Sitharaman B . Cytotoxicity, cytocompatibility, cell‐labeling efficiency, and *in vitro* cellular magnetic resonance imaging of gadolinium‐catalyzed single‐walled carbon nanotubes. J Biomed Mater Res A. 2013;101:3580‐3591. 23686792 10.1002/jbm.a.34643 PMC3785562

[72] Marangon I , Ménard‐Moyon C , Kolosnjaj‐Tabi J , et al. Covalent functionalization of multi‐walled carbon nanotubes with a gadolinium chelate for efficient T1‐weighted magnetic resonance imaging. Adv Funct Mater. 2014;24:7173‐7186.

[73] Tran LA , Krishnamurthy R , Muthupillai R , et al. Gadonanotubes as magnetic nanolabels for stem cell detection. Biomaterials. 2010;31:9482‐9491. 20965562 10.1016/j.biomaterials.2010.08.034 PMC2976808

[74] Gizzatov A , Hernández‐Rivera M , Keshishian V , et al. Surfactant‐free Gd^3+^‐ion‐containing carbon nanotube MRI contrast agents for stem cell labeling. Nanoscale. 2015;7:12085‐12091. 26119138 10.1039/c5nr02078f

[75] Chen C , Xing G , Wang J , et al. Multihydroxylated [Gd@C_82_(OH)_22_]_n_ nanoparticles: antineoplastic activity of high efficiency and low toxicity. Nano Lett. 2005;5:2050‐2057. 16218736 10.1021/nl051624b

[76] Liu Y , Jiao F , Qiu Y , et al. The effect of Gd@C_82_(OH)_22_ nanoparticles on the release of Th1/Th2 cytokines and induction of TNF‐α mediated cellular immunity. Biomaterials. 2009;30:3934‐3945. 19403166 10.1016/j.biomaterials.2009.04.001

[77] Tang AM , Ananta JS , Zhao H , et al. Cellular uptake and imaging studies of gadolinium‐loaded single‐walled carbon nanotubes as MRI contrast agents. Contrast Media Mol. 2011;6(2):93‐99. 10.1002/cmmi.410 21504063

[78] Servant A , Jacobs I , Bussy C , et al. Gadolinium‐functionalised multi‐walled carbon nanotubes as a T1 contrast agent for MRI cell labelling and tracking. Carbon. 2016;97:126‐133.

[79] Liu Y , Zhao Y , Sun B , Chen C . Understanding the toxicity of carbon nanotubes. Acc Chem Res. 2013;46:702‐713. 22999420 10.1021/ar300028m

[80] Dumortier H , Lacotte S , Pastorin G , et al. Functionalized carbon nanotubes are non‐cytotoxic and preserve the functionality of primary immune cells. Nano Lett. 2006;6:1522‐1528. 16834443 10.1021/nl061160x

[81] Chen Z , Liu Y , Sun B , et al. Polyhydroxylated metallofullerenols stimulate IL‐1β secretion of macrophage through TLRs/MyD88/NF‐κB pathway and NLRP3 inflammasome activation. Small. 2014;10:2362‐2372. 24619705 10.1002/smll.201302825

[82] Avti PK , Talukdar Y , Sirotkin MV , Shroyer KR , Sitharaman B . Toward single‐walled carbon nanotube–gadolinium complex as advanced MRI contrast agents: pharmacodynamics and global genomic response in small animals. J Biomed Mater Res B. 2013;101B:1039‐1049. 10.1002/jbm.b.32914 PMC3844433 23559429

[83] Boyles MSP , Stoehr LC , Schlinkert P , Himly M , Duschl A . The significance and insignificance of carbon nanotube‐induced inflammation. Fibers. 2014;2:45‐74.

[84] Goodarzi S , Da Ros T , Conde J , Sefat F , Mozafari M . Fullerene: biomedical engineers get to revisit an old friend. Mater Today. 2017;20:460‐480.

[85] Liu X , Gurel V , Morris D , et al. Bioavailability of nickel in single‐wall carbon nanotubes. Adv Mater. 2007;19:2790‐2796.

[86] Guo BJ , Yang ZL , Zhang LJ . Gadolinium deposition in brain: current scientific evidence and future perspectives. Front Mol Neurosci. 2018;11:335. 30294259 10.3389/fnmol.2018.00335 PMC6158336

[87] Choi JW , Moon W‐J . Gadolinium deposition in the brain: current updates. Korean J Radiol. 2019;20:134‐147. 30627029 10.3348/kjr.2018.0356 PMC6315073

[88] Tsoi KM , MacParland SA , Ma X‐Z , et al. Mechanism of hard‐nanomaterial clearance by the liver. Nat Mater. 2016;15:1212‐1221. 27525571 10.1038/nmat4718 PMC5132626

[89] Shultz MD , Wilson JD , Fuller CE , Zhang J , Dorn HC , Fatouros PP . Metallofullerene‐based nanoplatform for brain tumor brachytherapy and longitudinal imaging in a murine orthotopic xenograft model. Radiology. 2011;261(1):136‐143. 21813738 10.1148/radiol.11102569 PMC3176419

[90] Li J , Wang T , Feng Y , et al. A water‐soluble gadolinium metallofullerenol: facile preparation, magnetic properties and magnetic resonance imaging application. Dalton Trans. 2016;45:8696‐8699. 27064096 10.1039/c6dt00223d

[91] Jacobsen NR , Møller P , Clausen PA , et al. Biodistribution of carbon nanotubes in animal models. Basic Clin Pharmacol. 2017;121:30‐43. 10.1111/bcpt.12705 27865054

[92] Wang L , Luanpitpong S , Castranova V , et al. Carbon nanotubes induce malignant transformation and tumorigenesis of human lung epithelial cells. Nano Lett. 2011;11:2796‐2803. 21657258 10.1021/nl2011214 PMC3135732

[93] Principi E , Girardello R , Bruno A , et al. Systemic distribution of single‐walled carbon nanotubes in a novel model: alteration of biochemical parameters, metabolic functions, liver accumulation, and inflammation *in vivo* . Int J Nanomed. 2016;11:4299‐4316. 10.2147/IJN.S109950 PMC5012628 27621623

[94] Cui D , Tian F , Ozkan CS , Wang M , Gao H . Effect of single wall carbon nanotubes on human HEK293 cells. Toxicol Lett. 2005;155:73‐85. 15585362 10.1016/j.toxlet.2004.08.015

[95] Tang S , Tang Y , Zhong L , et al. Short‐ and long‐term toxicities of multi‐walled carbon nanotubes *in vivo* and *in vitro* . J Appl Toxicol. 2012;32:900‐912. 22760929 10.1002/jat.2748

[96] Deng X , Wu F , Liu Z , et al. The splenic toxicity of water soluble multi‐walled carbon nanotubes in mice. Carbon. 2009;47:1421‐1428.

[97] Singh R , Pantarotto D , Lacerda L , et al. Tissue biodistribution and blood clearance rates of intravenously administered carbon nanotube radiotracers. Proc Natl Acad Sci USA. 2006;103:3357‐3362. 16492781 10.1073/pnas.0509009103 PMC1413890

[98] Shu C , Corwin FD , Zhang J , et al. Facile preparation of a new gadofullerene‐based magnetic resonance imaging contrast agent with high 1H relaxivity. Bioconjugate Chem. 2009;20:1186‐1193. 10.1021/bc900051d PMC2862651 19445504

[99] Zhang J , Fatouros PP , Shu C , et al. High relaxivity trimetallic nitride (Gd_3_N) metallofullerene MRI contrast agents with optimized functionality. Bioconjugate Chem. 2010;21:610‐615. 10.1021/bc900375n PMC2862638 20218678

[100] Richard C , Doan B‐T , Beloeil J‐C , Bessodes M , Tóth É , Scherman D . Noncovalent functionalization of carbon nanotubes with amphiphilic Gd^3+^ chelates: toward powerful T1 and T2 MRI contrast agents. Nano Lett. 2008;8:232‐236. 18088153 10.1021/nl072509z

[101] Wang S , Zhou Z , Yu G , et al. Gadolinium metallofullerene‐polypyrrole nanoparticles for activatable dual‐modal imaging‐guided photothermal therapy. Acs Appl Mater Inter. 2018;10:28382‐28389. 10.1021/acsami.8b09670 30085649

